# Role of Adhesion Stress in Controlling Transition between Plastic, Grinding and Breakaway Regimes of Adhesive Wear

**DOI:** 10.1038/s41598-020-57429-5

**Published:** 2020-01-31

**Authors:** Andrey V. Dimaki, Evgeny V. Shilko, Ivan V. Dudkin, Sergey G. Psakhie, Valentin L. Popov

**Affiliations:** 1 Institute of Strength Physics and Materials Science SB RAS, pr. Akademicheskii 2/4., Tomsk, 634055 Russia; 2 Tomsk State University, Lenin ave. 36, Tomsk, 634050 Russia; 3 Technische Universität Berlin, Str. des 17. Juni 135, Berlin, 10623 Germany

**Keywords:** Mechanical engineering, Computational methods, Nonlinear phenomena

## Abstract

A discrete-element based model of elastic-plastic materials with non-ideal plasticity and with an account of both cohesive and adhesive interactions inside the material is developed and verified. Based on this model, a detailed study of factors controlling the modes of adhesive wear is performed. Depending on the material and loading parameters, we observed three main modes of wear: slipping, plastic grinding, cleavage, and breakaway. We find that occurrence of a particular mode is determined by the combination of two dimensionless material parameters: (1) the ratio of the adhesive stress to the pure shear strength of the material, and (2) sensitivity parameter of material shear strength to local pressure. The case study map of asperity wear modes in the space of these parameters has been constructed. Results of this study further develop the findings of the widely discussed studies by the groups of J.-F. Molinari and L. Pastewka.

## Introduction

Adhesive wear is a complex, multiscale and still poorly understood phenomenon^1–3^. This kind of wear occurs in contact pairs where materials of interacting bodies are characterized by equal or similar values of hardness with the possible transfer of material from one surface to the opposite one. Since the publication of Rabinowicz from 1958^4^, it is known that adhesive wear is controlled by the relation of material parameters including elastic modulus, hardness and specific surface energy^4,5^. In a series of recent works by Aghababaei, Molinari *et al*., it was shown that adhesive wear may develop in two ways: 1) formation and evolution (growth or fragmentation) of wear particles or 2) plastic deformation of micro contacts accompanied by smoothing of asperities^6,7^. These studies together with works by other research groups^8–11^ created a solid basis for a theoretical understanding of wear modes and opened possibilities for a new interpretation of classical concepts. In particular, the adhesive wear mode criterion by Rabinowicz was critically revised and details were disclosed of how the wear process occurs in both regimes of debris formation and plastic smoothing.

The above mentioned fundamental results revealing the features of asperity fracture and wear were obtained using pseudo molecular dynamics (MD) simulation at the mesoscale, so the question remained whether these conclusions are universal and applicable to real materials. A particular problem of meso MD models consists in the difficulty of separating the plastic and adhesive properties of material as in meso molecular dynamics both of them are determined by the same interaction potential. On the mesoscopic scale, it may be necessary, firstly, to decouple strength from adhesion parameters of the material and, secondly to take into account more complex material properties such as yield stress and strain hardening, both strongly dependent on features of internal structure at lower scales (grain structure, grain boundaries, inclusions of other phases, etc.). This substantiates the need for a detailed theoretical analysis of wear regimes using material model incorporating more details of material behaviour. In the present paper, we made an attempt to describe the details of adhesive wear process in the framework of a discrete element model with an explicit and independent account of plastic properties and of long-range adhesive forces acting between detaching parts of the bodies.

## Problem Statement

**Figure 1:**
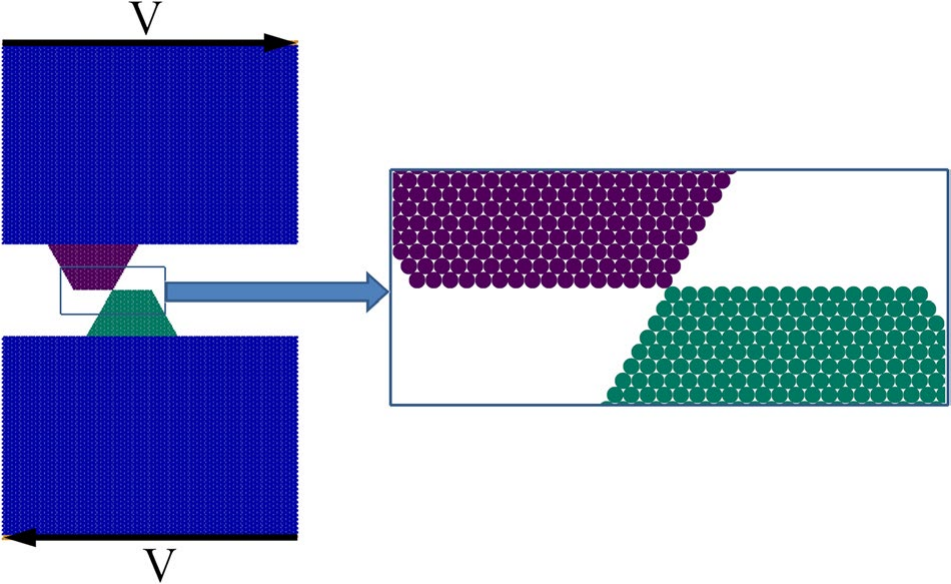
The structure of the model sample (fragment) and the scheme of loading.

The subject of the study is the contact of two asperities forced to collide with each other. We performed a numerical simulation in a plane strain approximation of an initially 3D problem using a Discrete Element Method (DEM) described in detail in “Materials and methods” section. Figure 1 shows the structure and the loading scheme of the considered system. Interacting asperities had equal size and trapezoidal shape – one of the typical simplified shapes used for generic studies of properties of “asperities” (see e.g.^11^). The height of asperity was about *D* = 0.5 mm (500 μm), the size of a discrete element was *d* = 0.025 mm (25 μm). The value of the interaction range of adhesive forces was δ = 1 nm (the detailed description of inter-element interaction is given in the “Materials and methods” section). Note that the main goal of the study was identifying the main governing parameters of the process, so the absolute values of the parameter used are of no primary importance. Additionally to the usual assumptions of DEM, we assumed that the parts of material interact with Dugdale type adhesive forces having final interaction range. The correctness of implementation of the adhesive interaction was verified by comparison with known analytical solutions for adhesive problems (see Supplementary Materials, Section 1).

We studied the interaction of asperities under the relative tangential motion of the surfaces at a constant velocity. The relative approach (overlap) of the upper and lower faces of the sample was kept constant and the bodies were forced to move with velocities *V* in opposite directions (see Fig. 1). On the left and right boundaries of the system, periodic boundary conditions were applied.

Due to the finite size of discrete elements and their regular close packing, the surfaces of asperities are regularly rough with the amplitude of roughness several times smaller than a discrete element size. The initial vertical positions of asperities we assigned to make them overlap by a distance between adjacent rows of close-packed elements $d/\sqrt{3}$. This overlap was sufficient for guaranteeing initiation of the processes of asperity welding with subsequent plastic deformation and fracture.

In the study, we assumed that direct contact of elements leads to the occurrence of chemical bonds (elements become “welded”). For separation, the Drucker- Prager condition was used (see Eq. (11) in the “Methods” section). This condition contains parameter *a*, which governs the brittleness of the material: it equals to 1 for highly ductile metals and polymers, while reaches 5–10 for elastic-brittle solids. The materials of the contacting bodies had equal physical-mechanical properties. We analyzed the influence of mechanical parameters of material (shear strength σ_*j*_, strength ratio *a*, yield stress σ_*y*_, strain hardening modulus *H* and Young’s modulus *E*) on the asperities wear mode. We varied the values of each mechanical parameter with respect to the “reference” value while all other parameters remained constant. There are the following “reference” values of mechanical characteristics: density ρ = 2000 kg/m^3^, Young’s modulus *E* = 1 GPa, Poisson’s ratio ν = 0.3, yield stress σ_*y*_ = 1 MPa, constant strain hardening coefficient *H* = 0.5 GPa (we assumed linear hardening law), σ_*c*_ = σ_*t*_ = 5 MPa (σ_*j*_ ≈ 2.89 MPa, *a* = 1). These values are of the order of magnitude of the values of the mechanical parameters of a number of polymers with elastic-plastic rheological properties.

## Numerical Analysis of the Regimes of Wear

For each assigned set of mechanical parameters, we carried out a series of numerical simulations for different values of Dugdale-like specific attractive force *σ*_0_ in the range from 0 up to the value of the material tensile strength σ_*t*_. We found that a realization of a particular wear mode of asperities is unambiguously determined by the values of material parameters. We revealed two most important factors determining a particular mode of asperity fracture.

1. The possibility of rebonding (restoration of integrity) of contact patches after they are pressed together. Introduction of rebonding results in the relative sliding consisting of a sequence of local bonding/debonding acts in the contact patches. Strength of newly bonded patches is characterized by pure shear strength and shear strength sensitivity to local pressure (the parameters σ_*j*_ and *a* respectively in fracture criterion (11)).
2. The presence of attractive normal interaction between spatially separated surfaces (“broken” or potential new contact patches). The simulation results show that changing the adhesion stress σ_0_ can change the wear mechanism.

The bonding-detachment process and “adhesion” characterize different stages of fracture process. When over critically loaded, first, chemical bonds have to be broken at the atomic scale, while much larger separation is needed to completely overcome the adhesive interaction.

The simulation allowed identifying several characteristic modes of interaction of asperities. We found that the transition from one mode to another is determined by a combination of the sensitivity parameter *a* and the ratio of attractive stress σ_0_ to the shear strength σ_*j*_.

We revealed two typical intervals of the values of the parameter *a* where different sets of wear modes are realized.

1. The interval 1 < *a* < 1.5 (typical for materials with high and moderate ductility).
2. The interval *a* > 1.5 (materials with limited ductility, quasi-brittle, and highly brittle materials).

In each of these intervals, the influence of dimensionless parameter *σ*_0_/σ_*j*_ has two sub-domains.

1. “Low” attractive forces: σ_0_ << σ_*j*_.
2. “High” attractive forces: σ_0_ ≥ σ_*j*_.

**Figure 2:**
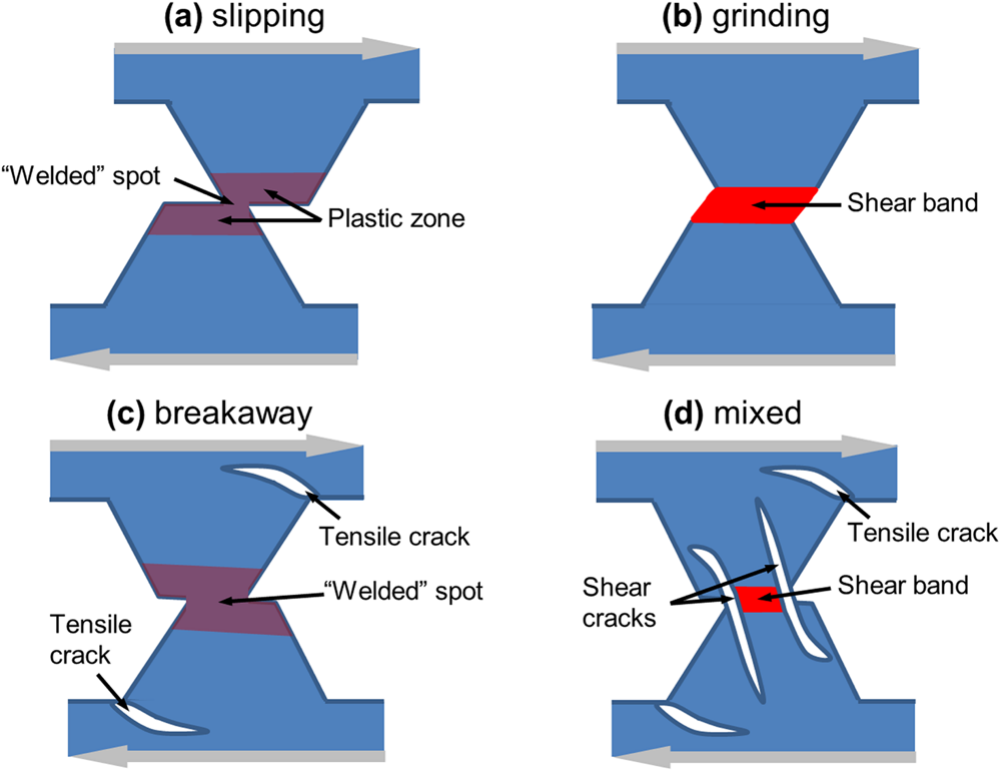
Typical wear modes of surface asperities under low-angle collision. Wear mode of ductile materials (*a* ≈ 1) transits from slipping (**a**) at σ_0_«σ_*j*_ to grinding-based fracture (**b**) at σ_0_ > σ_*j*_. The latter develops by means of “shear-band” layer formation and transfer of material adhered to one of the surfaces. More brittle materials (*a* > 1.5) wear by means of asperity detachment from the bulk (**c**) at σ_0_«σ_*j*_, while mixed “grinding-driven” mode (d) is typical at high adhesion σ_0_ > σ_*j*_.

Thus, the two-dimensional parameter space {*a*, σ_0_/σ_*j*_} can be subdivided into four domains. In each domain, a specific type of asperities interaction is realized (see conceptual illustration in Fig. 2). In the following, we consider in detail the main features of asperities wear in the above-mentioned ranges of the sensitivity parameter *a*.

### Wear of “ductile” asperities (a < 1.5)

Two qualitatively different modes of asperity interaction can be distinguished depending on the magnitude of attractive normal force σ_0_. They can be classified as “slipping” and “grinding” (or mixed “grinding” + ”cleavage”) modes.

(1) The first mode (“slipping”) is realized at ${\sigma }_{0} < {\sigma }_{0}^{\ast }$, where ${\sigma }_{0}^{\ast }$ is some critical adhesive force. In this case, asperities slip against each other without debris formation or significant damage of asperities. In this mode, sliding of the surfaces of interacting asperities is a sequence of relinking acts localized only in the contact plane. The asperities deform mainly elastically, and the small amount of plastic strain gradually accumulates in their volume (see Supplementary Materials, Section 2).

Note that multiple repetitions of asperities slipping can lead to their gradual smoothing or switching to the “grinding” mechanism discussed below. In addition, the “slipping” mode realization is only possible when a value of the asperities overlap is much smaller than an asperity height^12^.

(2) The second mode (“grinding-based” fracture) occurs at ${\sigma }_{0} > {\sigma }_{0}^{\ast } > {\sigma }_{j}$. In this mode, a fragmentation zone is formed where intensive processes of elastic energy dissipation are localized. There are two subdomains in the range *a* < 1.5, ${\sigma }_{0}\ge {\sigma }_{0}^{\ast }$, where “grinding-based” fracture leads to different degrees of fragmentation and wear.

**Figure 3:**
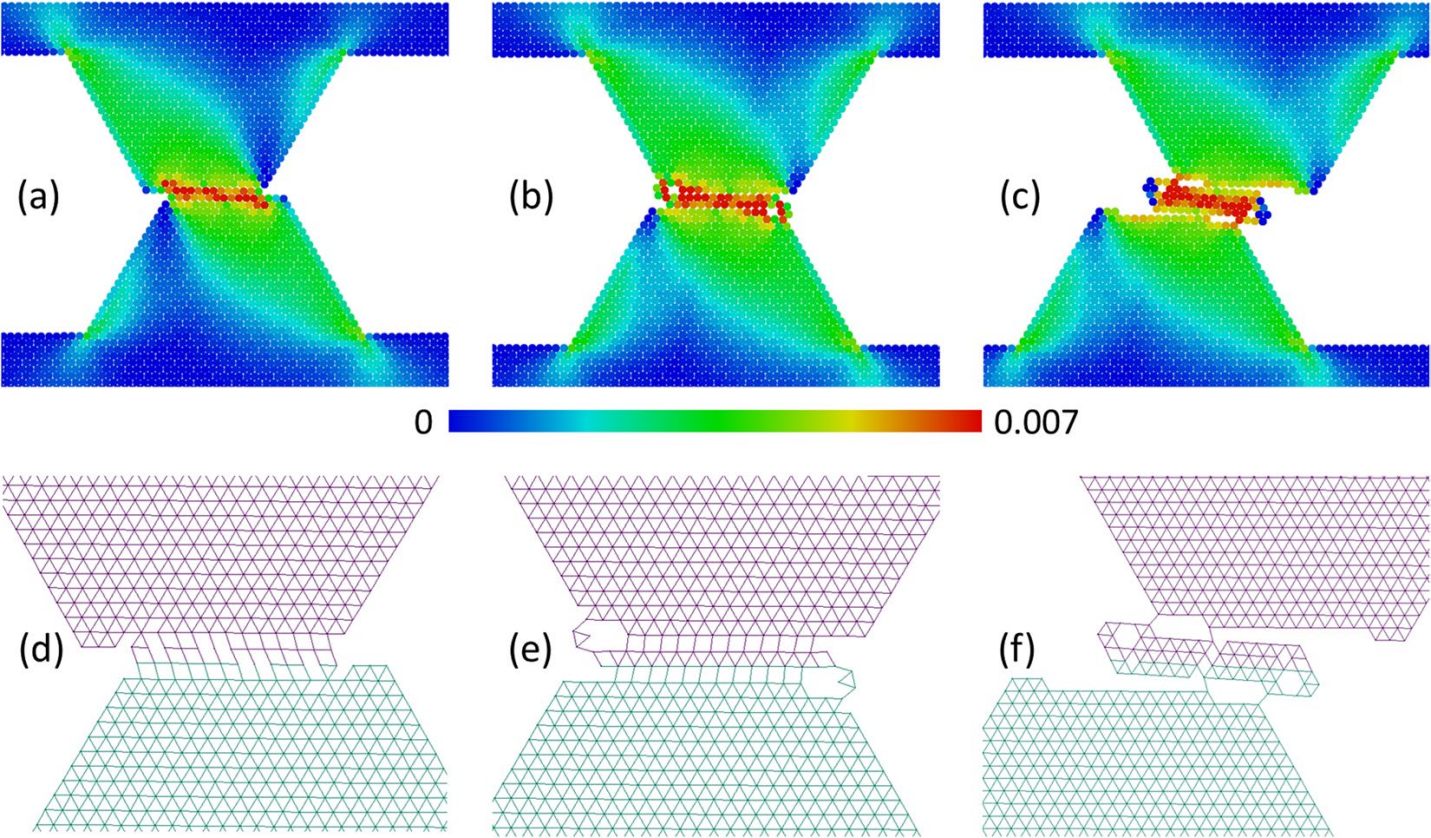
Example of “grinding” mode of asperity wear. The snapshots (**a–c**) show the structure and plastic shear strain ε_*xy*_ distribution in the asperities at consecutive moments during sliding. The snapshots (**d–f**) show chemically bonded (linked) discrete elements by line segments connecting their centers of mass. Adhesion parameters: σ_*j*_ = 4.5 MPa, *a* = 1, σ_0_ = 6 MPa. Other mechanical parameters have “reference” values.

In the range *a* < 1.2 (highly ductile materials), a gradual destruction of asperities within contact patches is realized by means of small wear particles separation (the size of these particles is much smaller than the size of asperity), sticking of these “debris” to an opposite surface, their transfer and so on (Figs. 3 and S5 in Supplementary Materials). Multiple local fractures and relinking occur in the contact plane and in the adjacent layers of material. This leads to a shear-band-like layer formation in a contact area, where processes of plastic deformation and energy dissipation localize. Indeed, a typical value of accumulated plastic shear strain in this region is several times larger than in “slipping” mode (see Figs. 3 and S3 in Supplementary Materials). A detachment of “large” (compared to asperity size) fragments of asperities does not take place in this mode, and multiple repetitions of the “grinding” can lead to a gradual attrition of interacting asperities. We call this grinding-based regime of fracture as “grinding”.

**Figure 4:**
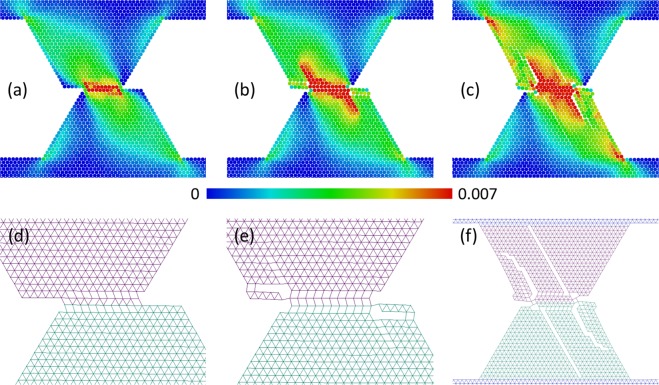
Example of mixed “grinding” + ”cleavage” mode of asperity wear. The snapshots (**a–c**) show the structure and plastic shear strain ε_*xy*_ distribution in the asperities at consecutive moments during sliding. The snapshots (**d–f**) show chemically bonded (linked) discrete elements by segments connecting their mass centers. Adhesion parameters: σ_*j*_ = 4.5 MPa, *a* = 1.3, σ_0_ = 6 MPa. Other mechanical parameters have “reference” values.

In the range *a* > 1.2 (moderately ductile materials) asperity destruction also begins from a “shear band” formation in the contact zone. However, when a contact area of asperities (the “shear band” length) becomes comparable to a size of a top facet of an asperity, cracks appear in the contact area and propagate into the bulk of asperities. These cracks lead to asperities splitting and to the separation of massive fragments (Fig. 4). We call this mode of fracture “grinding” + “cleavage”.

The described features of the grinding-based mechanism of asperity wear at different *a* originate from different pressure sensitivity of the shear strength. Since the “shear band” formation takes place under confined shear conditions, effective shear strength of this zone increases with an increase of the parameter *a*. At the same time, an absolute value of mean stress at the edges of the “shear band” is much lower than in the “shear band” central area. This leads to cracks initiation at the edges of the “shear band” if the value of *a* exceeds some threshold value.

There is a transient mode of asperity wear between “slipping” and “grinding-based” modes, that realizes at ${\sigma }_{0}\approx {\sigma }_{0}^{\ast }$. In this mode, the surfaces slide against each other similarly to the “slipping” mode (without “shear band” formation). At a late stage of sliding, a crack forms at the edge of the contact area and splits the asperity (“cleavage” mode). The pictures of this transient mode are shown in section 4 of Supplementary Materials. Note that a crack formation is due to high shear stresses at the edges of the contact area.

The obtained results demonstrate an important role of the attractive (adhesion) stress in the evolution of a contact area under confined shear. High attractive stress intensifies mass exchange between contact surfaces and thus facilitate “shear bands” formation. The magnitude of critical stress ${\sigma }_{0}^{\ast }$ separating the regions of two different mechanisms of asperity interaction depends on the values σ_*j*_ and *a* as well as on other material parameters (among them are yield stress, strain hardening coefficient, and Young’s modulus). These findings qualitatively well correlate with the results of molecular dynamics analysis of wear modes of nanoscopic asperities^11^. A clear boundary between atomic wear at low values of the work of adhesion (this can be considered as an analog of above-shown slipping mode) and much more intensive plastic wear at high values of the work of adhesion (analog of grinding-based mode) is theoretically shown in this paper. Similar to our case, the boundary value of the work of adhesion depends on the parameters of interatomic potential, which determine the material characteristics on the nanoscale.

### Wear of “brittle” asperities (a > 1.5)

As in the case of “ductile” materials, realization of specific mechanisms of asperity interaction for “brittle” materials is determined by the value of attractive force σ_0_. These mechanisms can be classified as “breakaway” and “grinding-based” (mixed “grinding” + ”breakaway”) modes.

**Figure 5:**
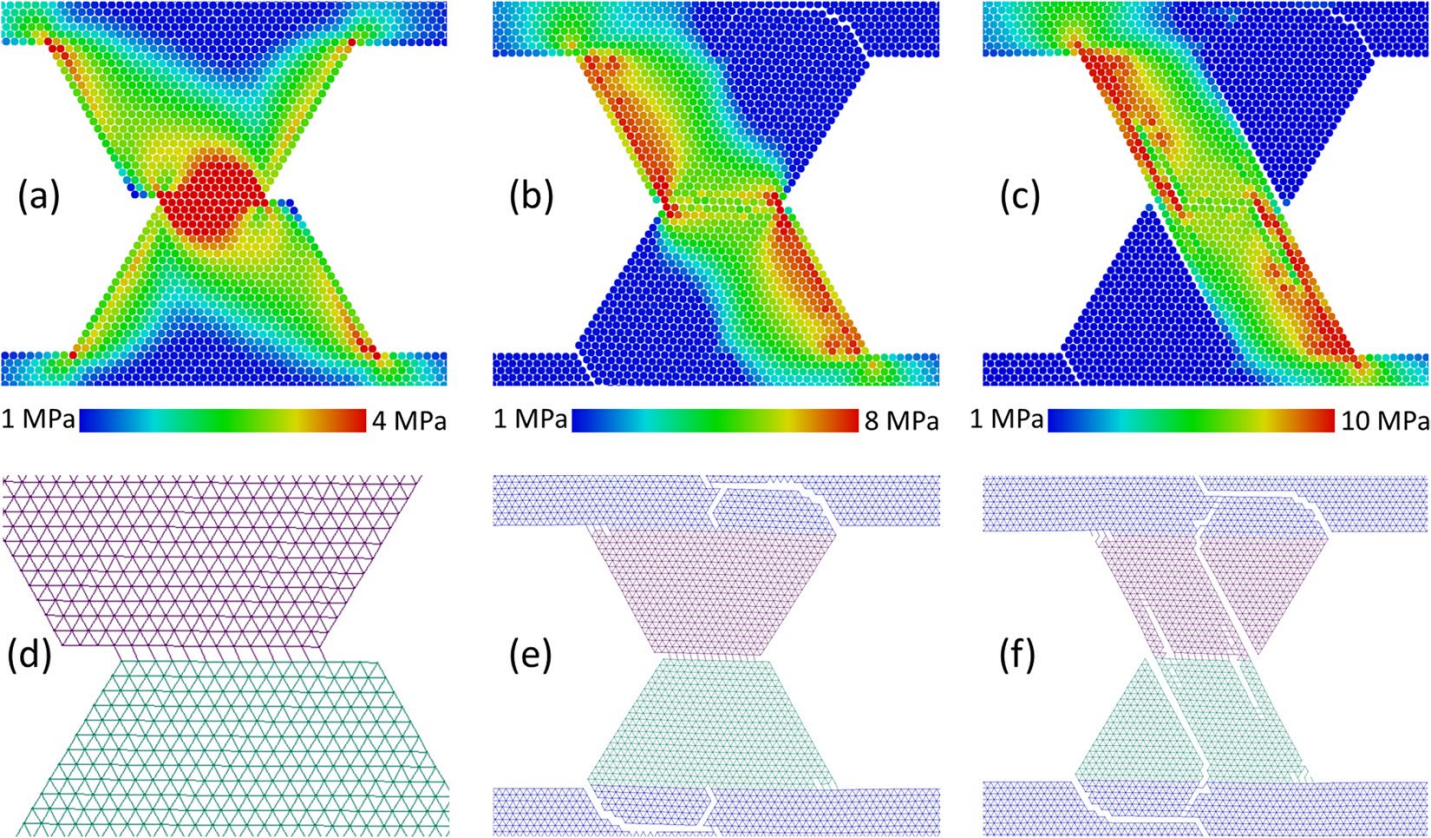
Example of “breakaway” mode of asperity wear. The snapshots (**a–c**) show the structure and equivalent stress σ_*eq*_ distribution in the asperities at consecutive moments during sliding. The snapshots (**d–f**) show chemically bonded (linked) discrete elements by segments connecting their mass centers. Adhesion parameters: σ_*j*_ = 4.5 MPa, *a* = 1.7, σ_0_ = 0 MPa. Other mechanical parameters have “reference” values.

(1) The “breakaway” mode realizes at ${\sigma }_{0} < {\sigma }_{0}^{\ast }$ by means of asperities separation from the contacting bodies and formation of debris particles (Fig. 5).

In this mode, surface interaction begins from slipping in the contact plane. Сontact area of asperities increases during sliding, thus tangential reaction force at the contact surface increases correspondingly. This force leads to asperities bending so that a frontal part of an asperity exerts tensile strain (stress distributions in the asperities are shown in Supplementary Materials, section 5). This leads to a tear crack formation in a bottom part of an asperity adjacent to the bulk of a main body.

**Figure 6:**
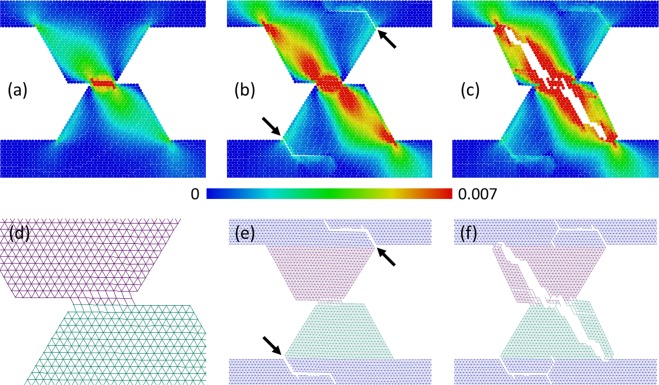
Example of mixed “grinding” + ”breakaway” mode of asperity wear. The snapshots (**a–c**) show structure and plastic shear strain ε_*xy*_ distribution in the asperities at consecutive moments during sliding. The snapshots (**d–f**) show chemically bonded (linked) discrete elements by segments connecting their mass centers. Adhesion parameters: σ_*j*_ = 4.5 MPa, *a* = 1.7, σ_0_ = 3 MPa. Other mechanical parameters have “reference” values.

(2) The “grinding” + “breakaway” fracture mode takes place at ${\sigma }_{0}\ge {\sigma }_{0}^{\ast }$ (Fig. 6). This mechanism is in many respects similar to the mixed mode of fracture of “ductile” asperities (see Section 3.1).

Asperity fracture begins from a shear-band-like layer formation in the contact zone (Fig. 6a,d). The contact area of asperities, “shear band” length and tangential reaction force increase during relative tangential motion. This is accompanied by an increase of a torque force and thus by an increase of tensile stress in the frontal part of an asperity. When approaching a critical contact area (that depends on *a* and does not exceed one half of a square of the top facet of an asperity), a tear crack forms in a frontal part of the asperity (the crack position is indicated by an arrow in Fig. 6b,e). Formation of this crack allows the asperities to turn, further resulting in fragmentation of asperities under constrained conditions (Fig. 6c,f).

## Discussion

The results shown in section 3 demonstrate the character of influence of parameters σ_0_, σ_*j*_ and *a* on the occurrence of a particular wear mode. We found that the critical value of attractive stress ${\sigma }_{0}^{\ast }$ is determined by the values of material parameters, particularly, by the value of pure shear strength. In order to study the mentioned dependence in more detail, we have carried out a series of numerical simulations with use of the “reference” values of mechanical parameters of the material (ρ, *E*, ν, σ_y_, *H*), but different values of strength parameters (σ_*j*_ and *a*). For each combination of strength parameters, we varied the value of attractive force σ_0_ from 0 up to σ_*t*_.

**Figure 7:**
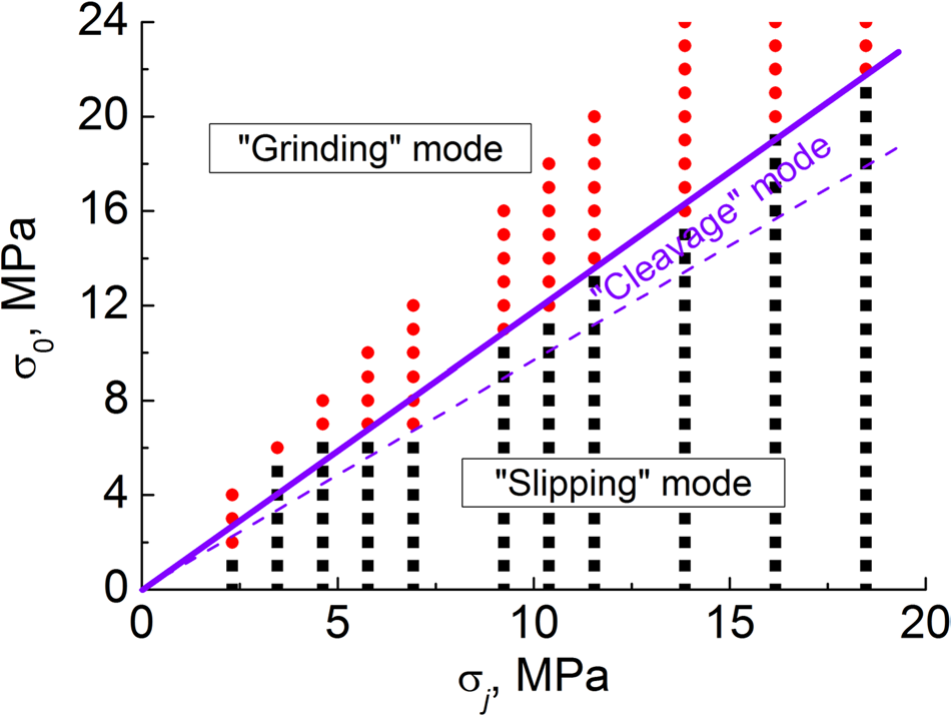
The “cloud” of points in the coordinate plane (σ_*j*_, σ_0_), where σ_*j*_ is the shear strength of the material, σ_0_ is the specific value of attractive force between spatially separated surfaces. Thick solid line limits the region of “grinding” wear mode from below. Dashed line delimits the regions where “slipping” and “cleavage” mechanisms of asperity interaction dominate. Red and black points correspond to “grinding” and “slipping”/”cleavage” modes respectively. “Fracture anisotropy” parameter was *a* = 1.0, “reference” values of elastic-plastic mechanical characteristics are used.

Figure 7 shows the “cloud” of points corresponding to samples with different values of pure shear strength σ_*j*_ and specific attractive force σ_0_. This particular example corresponds to von Mises strength criterion (*a* = σ_*c*_/σ_*t*_ = 1, ${\sigma }_{j}={\sigma }_{t}/\sqrt{3}$). The border values of σ_0_ separating the regions of “slipping” and “grinding” wear modes can be referred to as critical values ${\sigma }_{0}^{\ast }$. The physical sense of this rule is that the medium will plastically deform or separate depending on what is easier. If the adhesion stress is larger than the stress of plastic yielding, the medium will relax by plastic deformation. On the contrary, if the adhesion stress is smaller than the flow stress, it will separate. This rule strikingly reminds the old rule of Orowan-Ludwik-Davidenkov about the criterion for fully ductile, notch brittle and brittle behavior^13^.

The line separating the wear modes is well approximated by a straight line ${\sigma }_{0}^{\ast }={A}^{\ast }{\sigma }_{j}$. The empirical value of the coefficient of proportionality, A^*^, depends on the features of geometry of the asperity. In the considered case A^*^ ≈ 1.2. The analysis for “less ductile” materials (at *a* > 1) shows a similar trend with nearly the same proportionality coefficient A^*^. In general, the transition to the grinding-based wear mode takes place when the magnitude of specific attractive force σ_0_ is comparable to the pure shear strength of the material.

The numerically derived linear character of the dependence ${\sigma }_{0}^{\ast }({\sigma }_{j})$ in Fig. 7 well agrees with the following simple analytical estimation:

1 $${\sigma }_{0}^{\ast }={\rm{\beta }}(1+{\rm{\nu }}){\sigma }_{j}.$$

Here β is a dimensionless parameter depending on the material properties and the features of asperity geometry (the latter determine the features of stress distribution in the interacting asperities). The value of the parameter ${\rm{\beta }}={A}^{\ast }/(1+{\rm{\nu }})$ is close to 1 in the considered case.

Analytic estimation (1) has been derived based on the following considerations. The stress-strain state of the asperities near the contact patch can be characterized as “shear + compression”. In the vicinity of the contact patch, a plastic shear dominated deformation takes place. The possibility of large shear deformation during sliding of asperities is realized by multiple debonding and rebonding acts in different regions of the contact patch. Specific (per unit volume) value of elastic strain energy needed to realize an “act of sliding” under shear dominated loading can be estimated as ${E}_{el}^{shear}\approx {\sigma }_{j}^{2}/2G$.

During a relative tangential motion of the surfaces, interacting pairs of elements from opposite surfaces finally become non-contact after a link break. However, these non-contact elements are still attracted by the force σ_0_. Unlinked elements of approaching surface segments attract each other in the same way. At non-zero value of attractive force, this short-range interaction can provide healing (relinking of the broken bond) or formation of a new bond in closely spaced converging pairs. Therefore, an attraction between elements provides an effective “strengthening” of the contact surface and therefore promotes the accumulation of plastic strain and the formation of broken bonds in layers under the contact surface. This provides the condition for detachment of some fragments of these layers (or detachment of layers as a whole) from the asperity, their adhering to the opposite surface and transfer until a next relinking with their original asperity takes place.

The described factors generally facilitate a shear band formation in a contact zone. A similar effect for nanoscale asperities was extensively studied by molecular dynamics simulations^8,14,15^. Thus, the higher the attractive force σ_0_ the stronger is the tendency for strain localization and shear bands formation. Attractive force σ_0_ contributes to the stress-strain state of corresponding elements (it deforms an element and increases the equivalent stress and strain in its volume). A specific value of elastic strain energy of an element, concerned with the attraction of elements, can be estimated as ${E}_{el}^{attract}\approx {\sigma }_{0}^{2}/2E$.

Evidently, the realization of “grinding-based” modes of asperity wear is determined by the relation of two energies:

2 $$\Theta ={E}_{el}^{attract}/{E}_{el}^{shear}=({\sigma }_{0}^{2}/2E)/({\sigma }_{j}^{2}/2G) \sim {\sigma }_{0}^{2}/{\sigma }_{j}^{2}(1+{\rm{\nu }}).$$

In particular, higher values of ${E}_{el}^{attract}$ (i.e. higher values of the adhesion stress σ_0_) make the formation of “shear band” and implementation of the “grinding-based” regime of wear of asperity more energetically favorable. When Θ exceeds some critical value Θ_*crit*_, a transition from slipping/breakaway to grinding-based mode takes place. Θ_*crit*_ is a function of material parameters not included into (2):

3 $${\Theta }_{crit}=\frac{{({\sigma }_{0}^{\ast })}^{2}}{{\sigma }_{j}^{2}(1+{\rm{\nu }})}=f({\sigma }_{y},H,E,a).$$

Hence the linear dependence (1) follows as well as the fact that the coefficient β is a function of the parameters of the stress-strain curve and the sensitivity parameter *a* = σ_*c*_/σ_*t*_. Note that the given interpretation is applicable both for “ductile” and “brittle” materials. In both cases approaching some critical value of ${E}_{el}^{attract}$ leads to localization of plastic deformation in the contact area and to a more ductile pattern of asperity fracture than at relatively small ${E}_{el}^{attract}$.

The fundamental consequence of the relation (1) is that the condition for changing the mode of wear is not determined by the absolute value of specific attractive force σ_0_, but by the ratio of this force to the shear strength of the material. This result differs from the criterion obtained by Aghababaei *et al*.^6^ but is consistent with a later study^14^. The physical reason for this discrepancy may be the following: While the Rabinowicz-Aghababaei criterion^4–7^ considers the competition of the plasticity in the contact plane and separation in the region of stress concentration, our criterion considers the competition of plasticity and cracking directly in the region of a possible initial crack. If the critical “plastic stress” is smaller than the “adhesion stress”, then the cracking will not take place even if it is formally energetically allowed. The differentiation of the critical “plastic stress” and “adhesion stress” is related to some chosen plane in the material. Critical plastic stress is the tangential stress needed to initiate separation in some plane (plastic sliding of the surfaces of asperities or asperity detachment from the main part of the body). Critical “adhesion stress” is the normal component of stress which attracts separated surfaces in the chosen plane. The competition of plasticity and cracking at the same place (but in different planes) is well known both in molecular dynamics simulations and at the macroscale (as Orowan criterion). In a recent work by Brink and Molinari^14^, it was shown by direct three-dimensional quasi-molecular simulations that under some conditions the transition from one to another wear mode becomes “size insensitive”.

Note that parameters σ_*c*_ and σ_*t*_ used in the model for describing unlinking processes should be chosen mesh-dependent to provide mesh-independent macroscopic behavior^16^ (see details below in the Section “Methods”). However, their ratio *a* = σ_*c*_/σ_*t*_ is mesh-independent, and it is only this ratio which enters into the right part of the macroscopic criteria (3) for various wear modes.

It is well known that the value of macroscopic shear strength of the most of materials characterized by limited ductility (*a* > 1) depends on local mean stress^17^. Among these materials are cast irons, some intermetallic compounds, composites, ceramics, rock materials, etc.^18^. For these materials, key dimensionless parameters determining strength are pure shear strength σ_*j*_ and its sensitivity to the local mean stress *a = *σ_*c*_/σ_*t*_. Therefore, it is important to analyze the influence of this sensitivity parameter on the condition of the change of wear mode.

**Figure 8:**
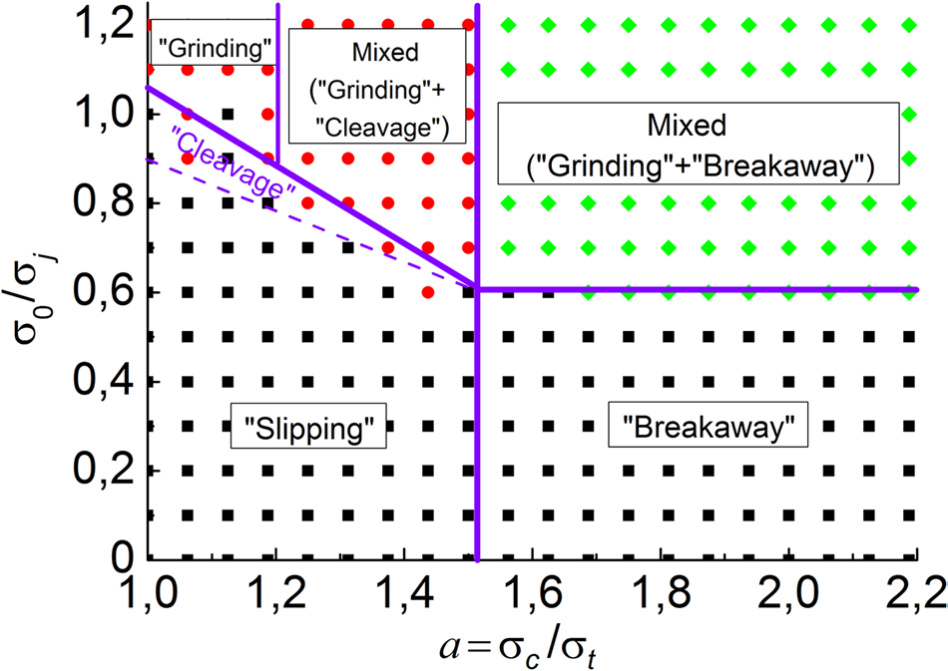
The case study map of asperity interaction modes in terms of two dimensionless parameters, which characterize adhesion. The cloud of points corresponds to different modes of wear under the variation of specific attractive stress σ_0_, material’s pure shear strength σ_*j*_ and the parameter *a* of the sensitivity of shear strength to mean stress. Purple lines delimit adjacent modes of asperity fracture. The “reference” values of elastic-plastic mechanical characteristics of the material are used.

We found that the critical value of attractive force (${\sigma }_{0}^{\ast }$), which delimits slipping/breakaway and grinding-based wear modes, is a non-linear function of the parameter *a*. Based on the simulation results at different values of adhesion parameters σ_*j*_, *a* and σ_0_, we obtained a case study map for the condition of low-angle asperity collision (Fig. 8). Note that specific numerical values of the boundary points (${\sigma }_{0}^{\ast }/{\sigma }_{j}$) have been obtained for “reference” values of elastic-plastic characteristics of the material and for trapezoidal interacting asperities.

Two intervals of anisotropy sensitivity parameter *a* are clearly seen in the map:

(1) For $a\in [1,\,1.5)$ that corresponds to moderately ductile materials (including cast irons, composites, etc.) and highly ductile ones (metals, plastics and so on), a transition from “slipping” to “grinding-based” wear mode takes place. The separation line between two regions is a linearly decreasing function of *a*. Here the parameter β is a linear function of *a*: ${\rm{\beta }}={{\rm{\beta }}}_{0}-{{\rm{\beta }}}_{1}(a-1)$, where the values of β_0_ and β_1_ are of the order of unity.

There is a transient mode of wear (“cleavage” of asperities) that takes place at σ_0_ slightly below the critical value ${\sigma }_{0}^{\ast }$. The width of the interval of the transient values of σ_0_, where the cleavage mode of fracture is implemented, is maximal for highly ductile materials (*a* = 1) and decreases to zero for more “brittle” materials (*a* ≥ 1.5).

The presented case study map helps to understand the peculiarities of the behavior of “highly ductile” and “moderately ductile” asperities. At ${\sigma }_{0} > {\sigma }_{0}^{\ast }$, highly ductile asperities demonstrate a pure “grinding” wear mode with the “shear band” formation in the contact zone. The shear band thickness determines the layer of material that can stick to one of the surfaces and be transferred by it, thus providing gradual smoothing/attrition of asperities. Wear of moderately ductile asperities also has grinding-based but more complex character. It also starts with “shear band” formation but then proceeds with cleavage of asperity. The reason for cleavage is the fact that high gradients of shear stress in asperity lead to local fracture due to a significant sensitivity of shear strength to mean stress. We refer this mode to as “grinding” + “cleavage”. The boundary (delimiting) value of the parameter *a* is about 1.2.

(2) For $a\ge 1.5$ that corresponds to materials with a “limited” ductility, semi-brittle and brittle, other wear modes are implemented. At low σ_0_, this is a “breakaway” mode. At ${\sigma }_{0} > {\sigma }_{0}^{\ast }$, a complex “grinding” + “breakaway” mode is realized (fracture starts with shear band formation but then proceeds with asperity detachment from the main body). The critical value ${\sigma }_{0}^{\ast }$ is independent of *a*. Corresponding value of the dimensionless coefficient β is $\beta \approx {\beta }_{0}-0.5{\beta }_{1}$.

The obtained results allow us to conclude that the basic idea of Rabinowicz^4^ about two dominating wear modes is applicable for materials with different sensitivities of shear strength to local pressure. An increase in the value of short-range attractive force σ_0_ leads to more “ductile” interaction and fracture of asperities. Since ${\sigma }_{0}^{\ast }$ depends on *a*, the criterion (1) has to be extended in order to take into account an influence of mean stress. We suggest the following empirical formulation of criterion (1):

4 $${\sigma }_{0}^{\ast }={\rm{\beta }}(1+{\rm{\nu }}){\sigma }_{j},\,{\rm{where}}\,\,\{\begin{array}{ccc}{\rm{\beta }}={{\rm{\beta }}}_{0}-{{\rm{\beta }}}_{1}(a-1) & {\rm{at}} & a < {a}_{crit}=1.5\\ {\rm{\beta }}={{\rm{\beta }}}_{0}-{{\rm{\beta }}}_{1}({a}_{crit}-1) & {\rm{at}} & a\ge 1.5\end{array}.$$

Equation (4) allows estimating the boundary between the “slipping/breakaway” and “grinding-based” modes of wear in a wide range of values of adhesion strength parameters.

## Conclusions

We developed and verified a contact model within the formalism of the discrete element method, which includes both plasticity and chemical bonding-debonding and adhesion with a finite range of action.

Using the developed model, we numerically studied the basic modes of asperities deformation and fracture including slipping, grinding/attrition, fragmentation, wear particle formation. The observed modes of interaction of asperities and involved mechanisms of asperity wear well agree with those reported for nanoscale asperities^8,14^. Two dimensionless parameters have been identified as especially important in determining the character of wear.

The most intriguing result is that the magnitude of the adhesion force between separated surfaces, along with the stress of plastic yielding and shear strength, controls the mode of fracture and thus the wear mode of asperities of different scales. Further, we observed and described a great variety of modes of wear, which were not described so far. However, all of them can be considered as modifications or transition phases of the fundamental limiting cases of plastic smoothing and breakaway which were described already by Rabinowicz. The results of the study are important for understanding the mechanisms of wear in tribological systems of different nature from traditional engineering tribological pairs to biological joints and tectonic faults.

## Materials and Methods

### Keystones of the numerical method

We used a discrete element (DE) approach based on the method of movable cellular automaton (MCA) described in a series of recent papers^19–23^. The formalism of the MCA method is based on the following assumptions.

(1) Discrete elements interact through the plane faces^24,25^ similar to the flat-joint model in classic implementations of DE method^26^. Normal and shear components of interaction forces are defined on the contact planes between elements. The initial value of the contact area is determined by the packing and size of discrete elements. In particular, in 2D problem formulation, the contact area of close-packed undeformed elements of the same equivalent radius *R* is equal to ${S}_{ij}^{0}=2Rh/\sqrt{3}$ (*h* is element size in the third dimension).

(2) When numerically solving equations of motion, the shape of a discrete element is approximated by equivalent sphere in 3D problem statement. This kind of implementation of DEM is called the distinct element method^25,27^. In the special case of elements motion in a plane (2D approximation) discrete elements are approximated by equivalent cylinders with the same height.

(3) The approximation of equivalent discs allows using simplified Newton-Euler equations of motion of a discrete element and separating an interaction between two elements into formally independent components, namely the central force (along the line connecting the mass centers of the elements) and tangential force (in the plane transverse to the mentioned line):

5 $$\{\begin{array}{l}{m}_{i}\frac{{d}^{2}{\overrightarrow{r}}_{i}}{d{t}^{2}}={m}_{i}\frac{d{\overrightarrow{v}}_{i}}{dt}=\mathop{\sum }\limits_{i=1}^{{N}_{i}}({\overrightarrow{F}}_{ij}^{n}+{\overrightarrow{F}}_{ij}^{\tau })\\ {J}_{i}\frac{d{\overrightarrow{\omega }}_{i}}{dt}=\mathop{\sum }\limits_{i=1}^{{N}_{i}}{\overrightarrow{M}}_{ij}\end{array},$$

where ${\overrightarrow{r}}_{{\rm{i}}}$, ${\overrightarrow{v}}_{i}$ and ${\overrightarrow{{\rm{\omega }}}}_{{\rm{i}}}$ are the three-dimensional radius-vector, the velocity vector and the pseudo-vector of angular velocity of element *i*, *m*_*i*_ is mass of the element *i*, *J*_*i*_ is moment of inertia of equivalent disc, ${\overrightarrow{F}}_{ij}^{n}$ and ${\overrightarrow{F}}_{ij}^{\tau }$ are central and tangential forces of interaction in *i-j* pair of elements, ${\overrightarrow{M}}_{ij}$ is the torque (moment of tangential force), *N*_*i*_ is the number of interacting neighbors. Specific values of interaction forces between elements *i* and *j* are:

6 $$\{\begin{array}{l}{\sigma }_{ij}={F}_{ij}^{n}/{S}_{ij}\\ {\tau }_{ij}={F}_{ij}^{\tau }/{S}_{ij}\end{array},$$

where *S*_*ij*_ is a contact area in *i*-*j* pair of elements.

(4) We assume homogeneous distributions of stresses and strains in the volume of an element (an approximation of simply deformable elements^22,27^). The stress-strain state of a discrete element is defined by average stress σ_αβ_ and average strain ε_αβ_ tensors. The components of these tensors are calculated through interaction forces, pair overlaps and relative shear displacements in the interacting pairs^21–23^. In particular, in the general 3D case, the component ${\sigma }_{\alpha \beta }^{i}$ of the average stress tensor in the volume of element *i* is determined as follows:

7 $${\sigma }_{\alpha \beta }^{i}=\frac{R}{{\varOmega }_{i}}\mathop{\sum }\limits_{i=1}^{{N}_{i}}{S}_{ij}^{0}[{\sigma }_{ij}{({\overrightarrow{n}}_{ij})}_{\alpha }{({\overrightarrow{n}}_{ij})}_{\beta }+{\tau }_{ij}{({\overrightarrow{n}}_{ij})}_{\alpha }{({\overrightarrow{t}}_{ij})}_{\beta }],$$

where α, β = *x*, *y*, *z* (XYZ is the laboratory coordinate system), Ω_*i*_ is the volume of element *i*, ${({\overrightarrow{n}}_{ij})}_{\alpha }$ and ${({\overrightarrow{t}}_{ij})}_{\alpha }$ are the projections of unit normal and unit tangent vectors onto the α-axis (unit normal vector ${\overrightarrow{n}}_{ij}$ is directed along the line connecting the mass centers of the interacting elements *i* and *j*, unit tangent vectors ${\overrightarrow{t}}_{ij}$ is directed in the tangential plane). The components of average strain tensor in the volume of element *i* are defined in the same way. In a special case of elements motion in a plane, the projections of unit vectors onto the third coordinate axis are zero.

(5) The assumption of a simply deformable element is implemented in the MCA using the original many-body formulation of the element-element interaction^22,23^. In particular, the specific central force of reaction of discrete element *i* in response to the action of element *j* consists of the pair-wise and pressure-dependent contributions, while tangential force is considered as a pair-wise:

8 $$\{\begin{array}{l}{\sigma }_{ij}={\sigma }_{ij}^{pair}({\varepsilon }_{ij})+{A}_{i}{\sigma }_{mean}^{i}\\ {\tau }_{ij}={\tau }_{ij}^{pair}({\gamma }_{ij})\end{array},$$

where the upper index “*pair*” denotes pair-wise function, which depends only on a relative displacement of the elements *i* and *j*, ${\sigma }_{mean}^{i}=({\sigma }_{xx}^{i}+{\sigma }_{yy}^{i}+{\sigma }_{zz}^{i})/3$ is a mean stress in the element *i*, *A*_*i*_ is a material parameter for the element *i*, ε_*ij*_ and γ_*ij*_ are normal and shear strains of the element *i* in the pair *i-j*. More comprehensive description of the features of the numerical method including the many-body formulation of inter-element interaction can be found in^22,23^.

(6) A local fracture is simulated as debonding of a pair of discrete elements. When debonding, state of a pair of discrete elements changes from “linked” to “unlinked” one. Here the term “linked” implies chemically bonded pair of elements that models consolidated fragment of material. The term “unlinked” means unbonded pairs of discrete elements (two independent fragments of a material). The condition of linked-to-unlinked transition is governed by a specified fracture criterion for a pair.

(7) Unlinked elements can become chemically bonded when coming into contact that is simulated by the transition from “unlinked” state of the pair to “linked” one. The condition of unlinked-to-linked transition is governed by a specified bonding criterion for a pair. The interaction between elements in a newly linked pair is the same as for originally linked pairs. The bonding of elements mimics “welding” of contacting surfaces. This effect is often taken into account in the modeling of contact interaction of clean and smooth surfaces.

### Keystones of the model of element-element interaction

In the present paper, we study adhesive contact interaction of the surfaces of isotropic linearly elastic and elastic-plastic bodies. In the latter case, we consider materials showing macroscopically inelastic response. The model of the interaction between elements is based on the following assumptions.

(1) Simulated material is isotropic. Mechanical behaviour of the material of a discrete element below the yield stress obeys the Hooke’s law:

9 $${\sigma }_{\alpha \beta }=2G{\varepsilon }_{\alpha \beta }+(1-2G/3K){\sigma }_{mean}{\delta }_{\alpha \beta },$$

where α, β = *x*, *y*, *z*; *G* and *K* are the shear and bulk elastic moduli of the material; σ_αβ_ and ε_αβ_ are components of average stress and average strain tensors; δ_αβ_ is the Kronecker delta. Within the plane strain approximation considered in the paper (motion of elements in XY plane and a fixed height of equivalent cylinders), the conventional condition ε_*zz*_ = ε_*xz*_ = σ_*xz*_ = ε_*yz*_ = σ_*yz*_ = 0 is applied to each discrete element.

The behaviour of the material of an element beyond the yield stress is simulated using the associated plastic flow model with von Mises yield criterion:

10 $${\sigma }_{eq}={\sigma }_{y},$$

where σ_*eq*_ is a value of equivalent stress in a discrete element (it is calculated with the use of the components of average stress tensor (7)); σ_*y*_ is the yield stress of the material of an element.

The plastic flow model is implemented using the radial-return algorithm of Wilkins^28^ specially adapted for a discrete element formalism. Details of the numerical implementation of elastic-plastic deformation of a discrete element can be found in^21–23^.

(2) Local fracture (linked-to-unlinked transition of a pair state) is described using local stress criterion applied to the linked pair (see details in^21–23^). We use the two-parametric failure criterion of Drucker and Prager^17^ in the following form^29^:

11 $${\sigma }_{eq}0.5(a+1)+{\sigma }_{mean}1.5(a-1)={\sigma }_{c},$$

where $a={\sigma }_{c}/{\sigma }_{t}$, σ_*c*_ is the uniaxial compressive strength of the material, σ_*t*_ is the uniaxial tensile strength that are input parameters of the fracture criterion. Here, equivalent stress σ_*eq*_ and mean stress σ_*mean*_ (negative hydrostatic stress) are invariants of the special stress tensor defined at the contact surface of two elements (details are provided in^21–23^). Note that the value of pure shear strength σ_*j*_ of the material (shear strength at zero confinement) can be easily derived from this criterion (${\sigma }_{eq}=\sqrt{3}{\sigma }_{j}$, ${\sigma }_{mean}=0$):

12 $${\sigma }_{j}=\frac{2{\sigma }_{c}{\sigma }_{t}}{\sqrt{3}({\sigma }_{c}+{\sigma }_{t})}=\frac{2{\sigma }_{c}}{\sqrt{3}(a+1)}=\frac{2{\sigma }_{t}a}{\sqrt{3}(a+1)}.$$

Criterion (11) takes into account the contribution of local volumetric stress to the condition of local fracture initiation in the linear approximation. The value of *a* reflects the brittleness of the material: it equals to 1 for highly ductile metals and polymers, while reaches 5–10 for elastic-brittle solids. Note that linear two-parametric failure criteria (Coulomb-Mohr, Drucker-Prager and so on) are often combined with Rankine (maximum principal stress) criterion in CAE software to better predict fracture under conditions of tension-dominated loading. In the present paper, we consider a shear-dominated loading with local contributions of volumetric tension and compression that is far from a tension-dominated stress state. Therefore we do not take into account tension cut-offs of the Drucker-Prager failure surface.

(3) Local bonding (unlinked-to-linked transition of the state of the pair) is taken into account in the model. We assume that the surfaces of discrete elements are not oxidized, chemically clean and atomically smooth (amplitude of roughness of a discrete element surface doesn’t exceed several crystal lattice parameters). Therefore, direct mechanical contact of unlinked elements surfaces leads to a chemical bonding between them: an unlinked pair becomes linked. This mimics “welding” of clean surfaces that takes place at microscopic and nanoscopic contact patches. Within this idealized model of chemical adhesion (cohesion), we assume that new bonds have the same strength as in the bulk of the bodies.

(4) One of the central points of this study is consideration of adhesion – attractive forces acting between elements even after they are debonded^30,31^. We use a simplified Dugdale’s interaction potential^32^: attractive normal stress is constant and equal to σ_0_ until the separation between the surfaces exceeds the distance δ (range of action of attractive potential). Thus the work of adhesion (effective surface energy), *w*_α_, is defined in the following simple manner: ${w}_{\alpha }={\sigma }_{0}\delta$. This simplified model allows reproducing a wide spectrum of “classic” adhesive theories including JKR^33^, DMT^34^ and a transition between them^35^.

The described attractive interaction between separated surfaces is implemented in the framework of the discrete element method in the following way. Two unlinked and not contacting discrete elements interact with constant attractive normal force *F*_*adh*_ if the distance between the surfaces of these elements does not exceed δ:

13 $$\{\begin{array}{l}{\sigma }_{ij}={\sigma }_{adh}={\sigma }_{0}\\ {F}_{ij}^{n}={F}_{adh}={\sigma }_{adh}S={\sigma }_{0}S\end{array},$$

where *S* is the contact area of interacting discrete elements. Specific attractive force σ_0_ is a material parameter for a given pair of interacting materials. The tangential force of interaction of unlinked and noncontact elements, which attract each other with a normal specific force σ_0_, is assumed to be zero. Note that the attractive normal force σ_0_ leads to tensile deformation of interacting elements along the line connecting their mass centers. Summarizing, unlinked and noncontact elements interact with constant attractive normal force (13) until they become non-interacting (if the distance between the surfaces exceeds δ) or linked (if the surfaces of the elements come into contact).

### A comment on the mesh dependency of mechanical properties

An important question of any numerical simulation based on a mesoscopic discrete element method is the “mesh dependency”: While the size of elements may be chosen somewhat arbitrary, the macroscopic behavior should not depend on this choice. In the present simulation technique, the criteria for unlinking the elements have to be chosen mesh-dependent to provide the macroscopic mesh size invariance. This fact was first shown in^36^ in the context of a related problem of adhesive strength considered in an adhesive crack propagation problem. The criterion suggested in^36^ was validated in^37^ by comparison with existing exact analytical solutions of adhesive problems and by direct comparison with specially designed experiments allowing observation of propagation of an adhesive crack. Later, the authors of the paper^16^ implemented this criterion in the method of Movable Cellular Automata and showed that, indeed, the introduction of the mesh-dependent “local fracture criterion” guarantees the mesh-independent behavior of the material.

The immediate implication of this “mesh dependency” of local fracture criteria is that dimensional parameters of these criteria do not rigorously correspond to macroscopic material property. Therefore, these dimensional parameters may not appear in criteria describing the physical behavior of the real system. In the present model of fracture, the mesh-dependent critical quantities are σ_*c*_ and σ_*t*_. Both have the same mesh dependency but their ratio is mesh independent. Only this ratio may enter any macroscopic physical criterion, which exactly is the case in our study of the conditions of asperities wear mode change. This inference is supported by the special study of mesh dependence of the boundary between qualitatively different regimes of asperity wear: plastic smoothing at “high” adhesion stresses and slipping/cleavage at “low” adhesion stresses (see Supplementary Materials, Section 7). Thus, we conclude that our results generalized in terms of dimensionless combinations of adhesion parameters are mesh size invariant.

## Supplementary information

Supplementary Materials

## Data Availability

All data generated or analysed during this study are included in this published article and its Supplementary Information file.
