# Supplementary material for: Role of Adhesion Stress in Controlling Transition between Plastic, Grinding and Breakaway Regimes of Adhesive Wear
Source: Sci Rep. 2020 Jan 31;10:1585. doi: 10.1038/s41598-020-57429-5 (PMC6994689; doi:10.1038/s41598-020-57429-5)
Supplement: Supplementary file 1 — Supplementary Materials [file 41598_2020_57429_MOESM1_ESM.docx]

Supplementary Materials for

**Role of Adhesion Stress in Controlling Transition between Plastic, Grinding**

**and Breakaway Regimes of Adhesive Wear**

Andrey V. Dimaki ^1^,*, Evgeny V. Shilko ^1,2^, Ivan V. Dudkin ^1^, Sergey G. Psakhie ^1^

and Valentin L. Popov ^2,3, *^

^1^Institute of Strength Physics and Materials Science SB RAS, pr. Akademicheskii 2/4., Tomsk 634055, Russia

^2^Tomsk State University, Lenin ave. 36, Tomsk 634050, Russia

^3^Berlin University of Technology, str. des 17 Juni 135, Berlin 10623, Germany

*****Correspondence: dav@ispms.tsc.ru and v.popov@tu-berlin.de

1. **Verification of the implementation of adhesive interaction**

Implementations of the described DEM-based model having a zero inter-element attractive force (σ_0_=0 and δ=0) were previously verified and successfully applied to study the features of brittle and/or ductile materials friction [1,2]. On the contrary, the adhesive contact model with Dugdale-like approximation of the attractive force between separated elements is firstly proposed in the present paper. Therefore, it is necessary to check the correctness of the numerical implementation of the Dugdale-like adhesive interaction of the surfaces.

We have proven the correctness of the model by means of comparison of the results of numerical calculations with known analytical solutions for normal frictionless contacts [3,4]. We considered a normal contact of a rigid cylinder and an elastic half-space (see Figure S1). The cylinder and the body were modeled by an ensemble of close-packed discrete elements.

| 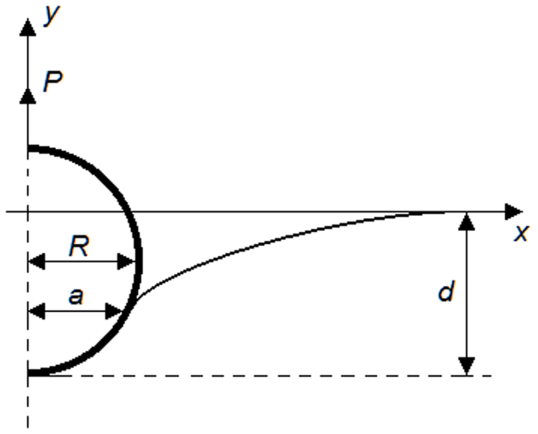 |
| --- |

Figure S1. Scheme of a cross-section of a long rigid cylinder with a radius *R* indented into an isotropic purely elastic half-space.

In the framework of the verification procedure, we did not take into account fracture and chemical bonding. Also, we assigned zero value of the coefficient of friction in unlinked and contact pairs of discrete elements belonging to interacting surfaces (only normal interaction between these elements was taken into account). These simplifications were necessary to perform a quantitative comparison between simulation results and the classical analytical solution obtained for a frictionless normal contact.

In the simplest case of normal purely elastic contact without adhesion, the dependence of a specific reaction force on a contact radius *a* reads

$P=\frac{\pi E^{*}a^{2}}{R}$, (S.1)

where $E^{*}=E/\left(1-\upsilon^{2}\right)$ – is an effective elastic modulus of the half-space in plane strain conditions, *a* – is a half-width of the contact area, *R* – is the radius of the rigid cylinder. The dependencies *P*(*a*) obtained from a numerical calculation as well as the same given by Eq. (S.1), are shown in Figure S2a. There is a good agreement between the results of numerical DEM simulation and the corresponding analytical solution for a non-adhesive contact.

| 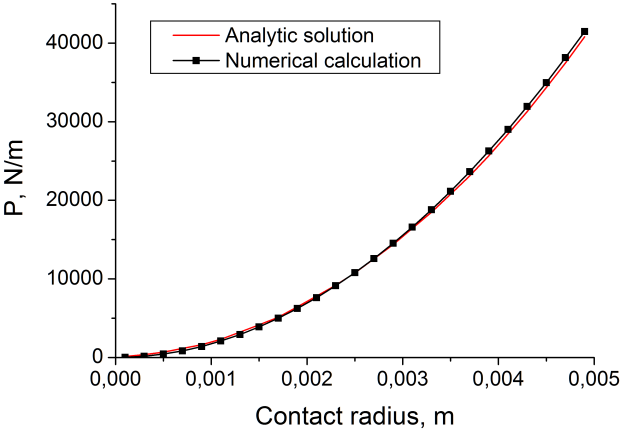  (**a**) | 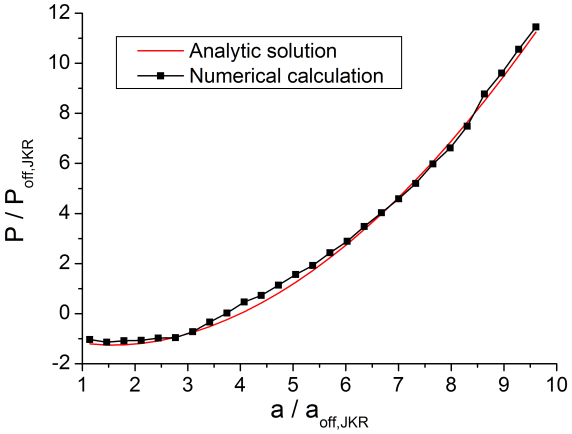  (**b**) |
| --- | --- |

Figure S2. The dependencies of the specific reaction force (per unit length) on the contact radius for a rigid cylinder indented into an elastic half-space: (**a**) Contact without adhesion; (**b**) Contact with adhesion.

Assuming the presence of adhesive forces and that the surface energy of the materials of the cylinder and the half-space is Δγ, a specific line reaction force in the contact is given by [5]:

$P=\frac{\pi E^{*}a^{2}}{R}-2\sqrt{2\pi E^{*}a\Delta\gamma}$. (S.2)

According to the results of JKR theory, the specific pull-off load needed to detach the cylinder from the half-space is

$P_{off,JKR}=-3\pi E^{*}R^{1/3}\left(\frac{\Delta\gamma}{2\pi E^{*}}\right)^{2/3}$. (S.3)

The contact radius at pull-off is

$a_{off,JKR}=R^{2/3}\left(\frac{\Delta\gamma}{2\pi E^{*}}\right)$. (S.4)

We carried out a numerical simulation of contact between a cylinder and a half-space in the framework of the developed DEM model. Note that in the present calculation the value of the Maugis’ parameter

$\lambda=\frac{2\sigma_{0}}{\left(16\pi\sigma_{0}\left(E^{*}\right)^{2}/9R\right)^{1/3}}$ (S.5)

is λ≈20 that indicates the JKR-like character of the behavior of the contact.

The dependence of the specific normal reaction force normalized to $P_{off,JKR}$ on the contact radius normalized to $a_{off,JKR}$ is shown in Figure S2b. It is seen that the dependence *P*(*a*), obtained in the developed discrete-element model, is in a good agreement with the corresponding analytical dependence (S.2).

The results shown above demonstrate the correctness of the developed DEM-based model of an adhesive interaction and allow us to apply this model to study the regimes of wear with taking into account attractive forces.

1. **The “slipping” mode of asperities interaction**

Figure S3 shows a typical example of accumulation of plastic shear strain ε*_xy_*. Maximum shear strain is localized under the contact zone. Note that the maximum magnitude of elastic shear strain in the asperities during the course of contact interaction is 2-3 times larger than accumulated inelastic value.


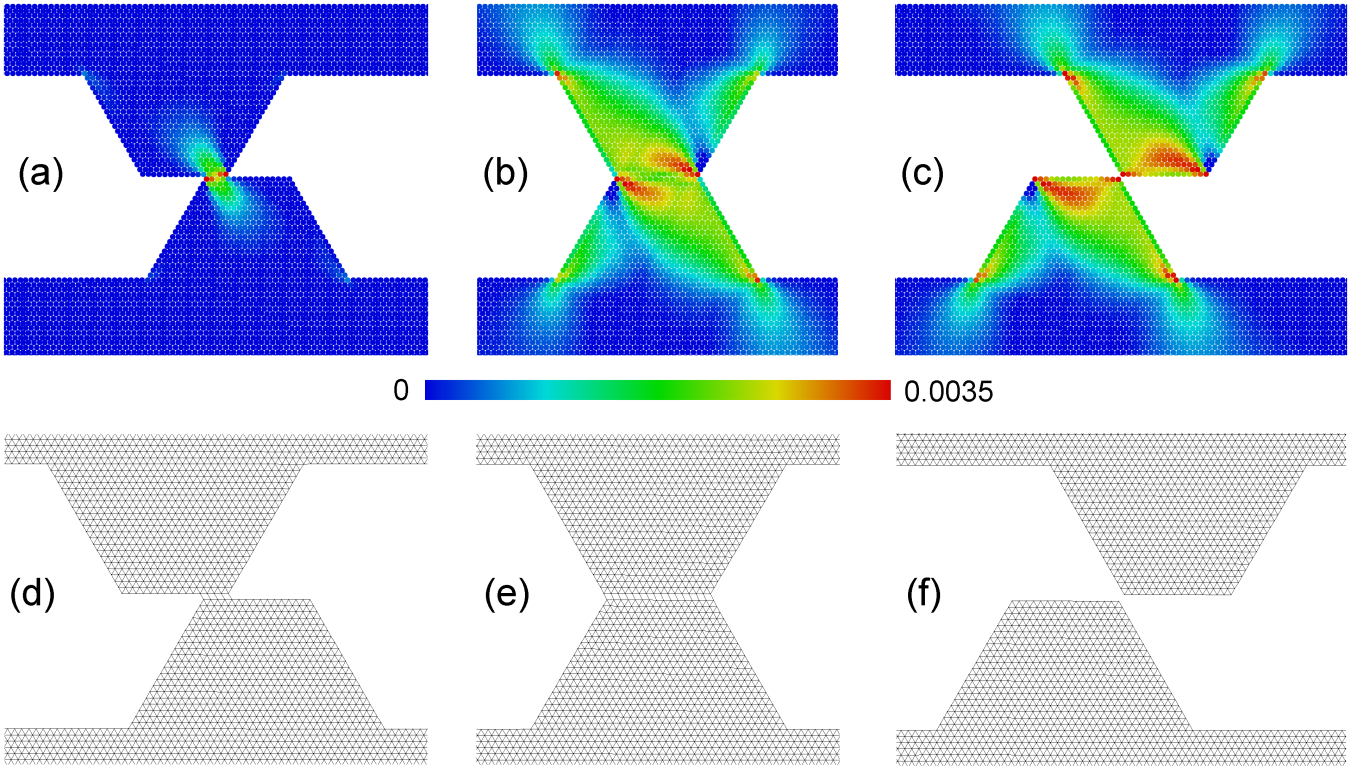


Figure S3. “Slipping” mode of interaction of asperities. The snapshots (a)-(c) show the structure and plastic shear strain ε*_xy_* distribution in the asperities at consecutive moments during sliding. The snapshots (d)-(f) show chemically bonded (linked) discrete elements by line segments connecting their mass centers. Adhesion parameters: σ*_j_*=4.5MPa, *a*=1, σ_0_=1MPa. Other mechanical parameters have “reference” values.

The shown example corresponds to relatively high values of pure shear strength σ*_j_* exceeding the “reference” value. At a high σ*_j_*, sliding is not accompanied by damage accumulation. At lower magnitudes of pure shear strength including the “reference” value σ*_j_*≈2.89 MPa, sliding is accompanied by the formation of individual damages at the contact surfaces (Figure S4). Nevertheless, wear realized by element-by-element (or layer-by-layer) attrition or fracture of an asperity doesn’t take place.


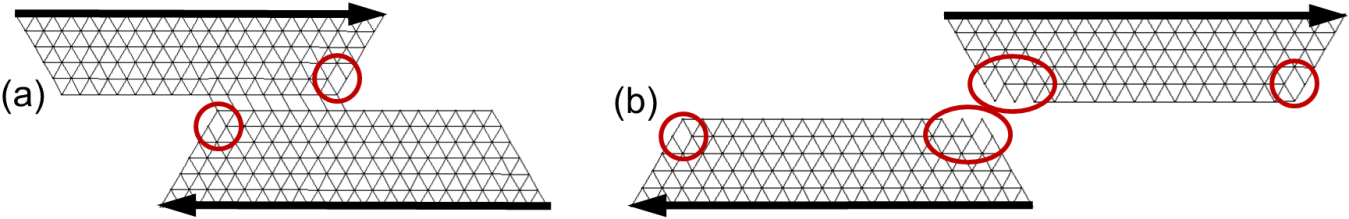


Figure S4. Formation of damages (broken chemical bonds) during relative tangential motion of asperities. The contact zone and underlying region of asperities at intermediate (a) and final (b) stages of sliding. The black arrows show the direction of motion of the contacting bodies. The red ovals enclose damages. Adhesion parameters: σ*_j_*=2.89MPa, *a*=1, σ_0_=1MPa. Other mechanical parameters have “reference” values.

Detailed analysis of this mechanism by means of molecular dynamics simulations [6,7] shows that slipping is accompanied by wear effects on the atomic scale in the form of sticking of one or several atomic layers to the opposite surface and their transfer. These effects are noticeable only for nanoscale roughness [6,7] and therefore not discussed in this paper. Nevertheless, multiple repetitions of this mode of asperities interaction can potentially lead to their gradual smoothing or involvement of the “grinding” mechanism discussed below.

1. **Example of wear evolution in “grinding” mode**


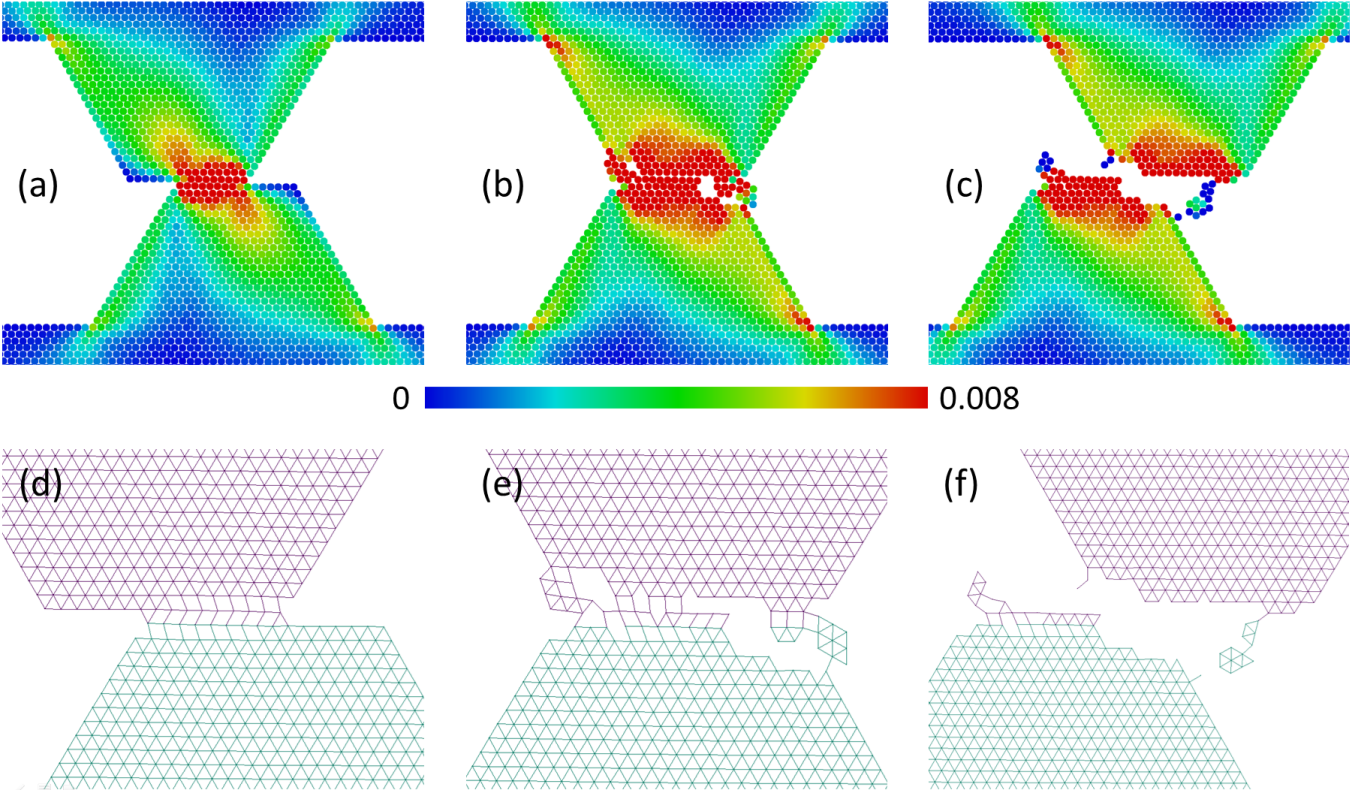


Figure S5. Example of “grinding” mode of asperity wear. The snapshots (a)-(c) show the structure and plastic shear strain ε*_xy_* distribution in the asperities at consecutive moments during sliding. The snapshots (d)-(f) show chemically bonded (linked) discrete elements by line segments connecting their mass centers. Adhesion parameters: σ*_j_*=7MPa, *a*=1.1, σ_0_=11MPa. Other mechanical parameters have “reference” values.

1. **Examples of wear evolution in “cleavage” mode**


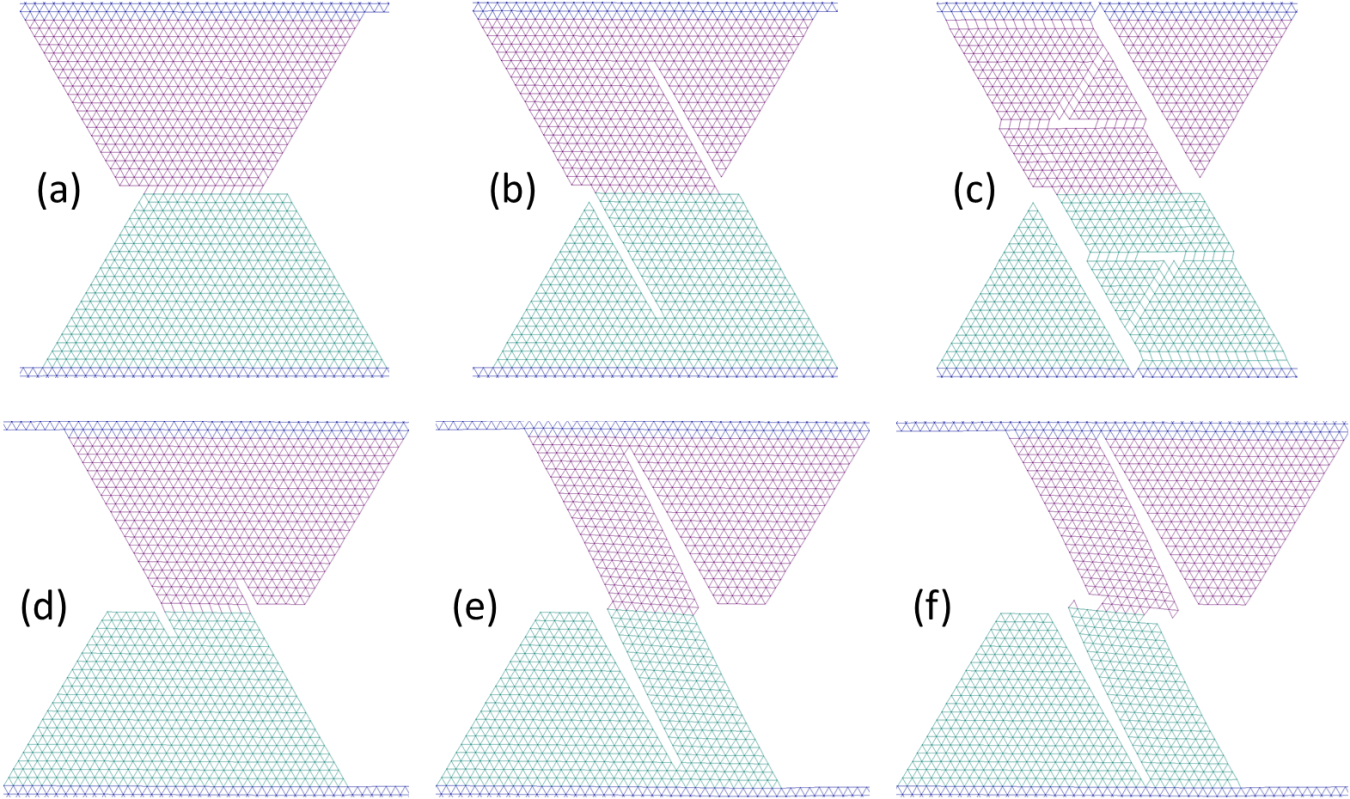


Figure S6. Examples of “cleavage” mode of asperity wear: (a)-(c) σ*_j_*=2.89MPa, *a*=1.0, σ_0_=2.6MPa; (d)-(f) σ*_j_*=7MPa, *a*=1.3, σ_0_=5.7MPa. Other mechanical parameters have “reference” values. The snapshots show chemically bonded (linked) discrete elements by segments connecting their mass centers.

1. **Stress distributions the contact area in the “breakaway” mode of wear**

In the process of the relative tangential movement (sliding) of asperities, the tangential resistance force at the contact surface gradually increases. This force is torque force, that is, causes bending of the asperity. At that, the frontal part of the base of the asperity undergoes a tensile strain, while the rear part of the base undergoes a compressive strain (Figure S7).


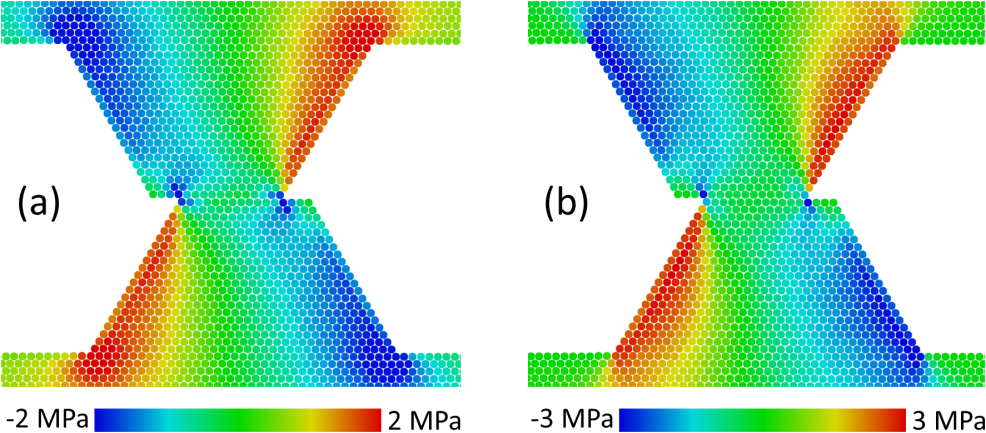


Figure S7. Distributions of mean stress σ*_mean_* (a) and diagonal component σ*_yy_* of the stress tensor (b) in the asperities just before the beginning of asperity tearing off the main body. Adhesion parameters: σ*_j_*=4.5MPa, *a*=1.7, σ_0_=0MPa. Other mechanical parameters have “reference” values. The shown snapshots correspond to the snapshots in Figures 5a and 5d of the main body of the paper.

For materials with high sensitivity of shear strength to local pressure, an effective strength of a contact area under confined shear is significantly higher than an effective strength of a frontal part of an asperity base (the latter is under the condition of shear+tension). This leads to a tear crack formation in a bottom part of an asperity adjacent to the bulk of the main body (Figures 5b and 5e in the main body of the paper). While a crack propagates to a rear part of an asperity base, this crack comes into a region under shear and compression loading conditions (Figure S7). The mentioned change of stress state leads to suppression of the crack growth and further crack motion into the bulk of the asperity as a shear crack. It is evident from the present description that the “breakaway” mode of wear can realize in materials with a strong dependence of shear strength on local pressure.

1. **Influence of the plasticity parameters of material on the wear mode**

We have carried out a numerical study to analyze the influence of the plasticity parameters of material on the mechanisms of asperity interaction and the condition of changing the wear mode (the boundary value $\sigma_{0}^{*}$ of attractive force).

The key parameters of material plasticity in the framework of the used model with von Mises yield criterion are strain hardening modulus and yield stress. In the analytical model of Rabinowicz [8], the ideal plasticity of materials has been postulated. In the paper, we studied the effect of the value of strain hardening modulus in order to estimate limits of applicability of the concept of two dominating regimes of wear for materials with non-ideal plasticity. We varied the value of strain hardening modulus from 10% up to 100% of Young’s modulus at fixed values of other material parameters. It was revealed that the critical value of attractive force $\sigma_{0}^{*}$ doesn’t depend on strain hardening modulus up to *H*≈0.75*E*. At that, the mechanisms of asperity interaction do not change with *H* (Figure S8).


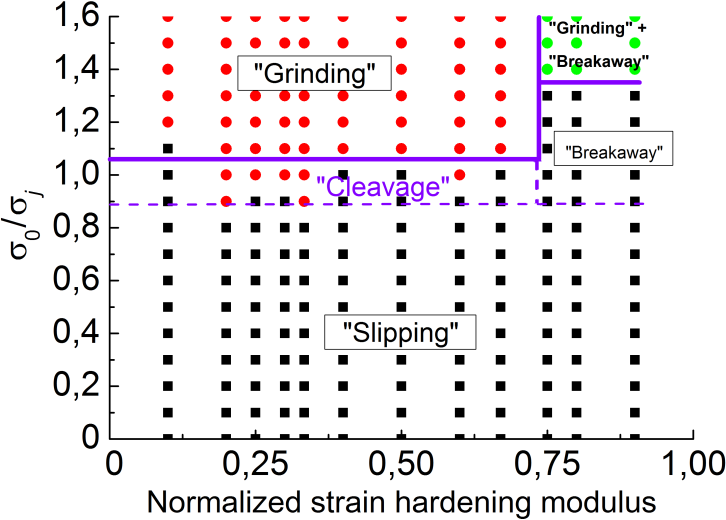


Figure S8. The case study map of asperity interaction modes in terms of two dimensionless material parameters: the ratio of specific attractive force $\sigma_{0}^{*}$ to material’s pure shear strength σ*_j_* (the ordinate axis) and strain hardening modulus *H* normalized to Young's modulus *E* (the abscissa axis). The map corresponds to materials with von Mises strength (*a*=1) and “reference” values of Young’s modulus and yield stress.

Figure S9 supports the above thesis about the same type of the features of asperity interaction at different values of strain hardening modulus below the threshold *H*≈0.75*E*. This figure shows two typical modes of asperity interaction (“slipping” and “grinding”) for the case of very low strain hardening modulus (*H*/*E*=0.05). Comparison with Figure 3 (main body of the paper), Figure S3 and Figure S5 shows that although a change in strain hardening modulus by an order of magnitude leads to a strong localization of irreversible strains near the contact zone of asperities, it does not change qualitatively the pattern of strain distribution and the mode of asperity interaction. In particular, the process of inelastic strains accumulation in the asperities without debris formation takes place at small adhesion stresses σ_0_. At large adhesion stresses, the interaction of asperities is shear band driven. Gradual destruction of asperity is realized by means of small wear particles separation (the size of these particles is much smaller than the size of asperity), sticking of these “debris" to an opposite surface, their transfer and so on.


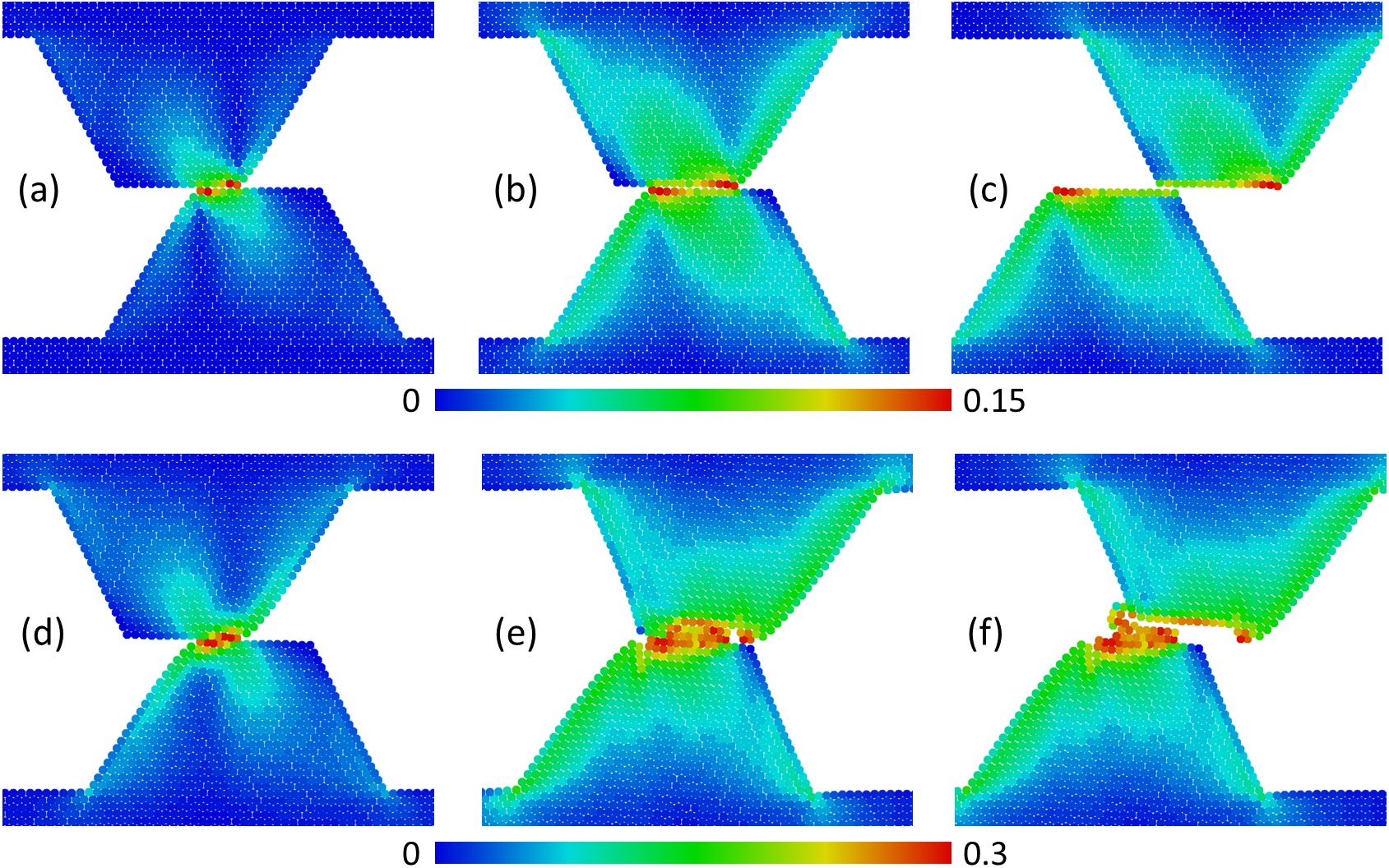


Figure S9. Examples of “slipping” (a)-(c) and “grinding” (d)-(f) modes of asperity interaction for the case of “very low” values of strain hardening modulus *H* (*H*/*E*=0.05). The upper row of snapshots shows the structure and equivalent plastic strain ε*_eq_* distribution in the asperities at consecutive moments during sliding at “small” adhesion stress σ_0_= 1MPa, while the lower row corresponds to “high” adhesion stress σ_0_= 11MPa. Strength parameters of the material are the same for both cases: σ*_j_*=7MPa, *a*=1.0. Other mechanical parameters have “reference” values.

For higher values of strain hardening modulus (*H*>0.75*E*) the character of interaction of asperities changes (Figure S8). While at low σ_0_ the mechanism of asperity interaction is the same as in the region *H*<0.75*E*, at $\sigma_{0}>\sigma_{0}^{*}$ the character of asperity wear becomes “more brittle”. While the value of σ_0_ increases, the wear mode changes from “slipping” to “breakaway” and further (at $\sigma_{0}>\sigma_{0}^{*}$) to “grinding”+”breakaway”. The value of $\sigma_{0}^{*}$ in the region *H*>0.75*E* is up to 1.5 times higher than at *H<*0.75*E* (this rule is common for different *a*).

Variation of the yield stress of the material influences the transition between wear modes in a similar manner as the variation of strain hardening modulus. Under relatively low values of yield stress up to 30-35 percent of the tensile strength of the material, a variation of the yield stress doesn’t influence the mechanisms of asperity interaction. The proposed equation (4) in the main body of the manuscript for critical attractive force determining the border between two wear modes includes shear strength σ*_j_* in contrast to the original model of Rabinowicz where yield stress is used [8]. This is due to the fact that we consider materials with non-ideal plasticity for which the ultimate stress is not limited by the yield stress. Note that Aghababaei and Molinari et al. [9,10] proposed a modified formulation of Rabinowicz’s criterion that also includes shear strength.

From this point of view, it is clear that yield stress doesn’t determine a transition between wear regimes and thus does not enter criterion (4) at σ*_y_*<0.3-0.35σ*_t_*.

Just like in the previous case, a character of asperity fracture significantly changes in the region σ*_y_*>0.3-0.35σ*_t_*. While at $\sigma_{0}<\sigma_{0}^{*}$ the mechanism of asperity interaction doesn’t change in comparison with the materials with low σ*_y_*, at $\sigma_{0}\geq\sigma_{0}^{*}$ it is generally more “brittle”. Increase in the values of σ_0_ is accompanied by an unsystematic change in asperity fracture pattern. In particular, for the case *a*=1 all above-mentioned wear modes are implemented: “pure grinding”, “grinding”+”cleavage” and “grinding”+”breakaway” (Figure S10).


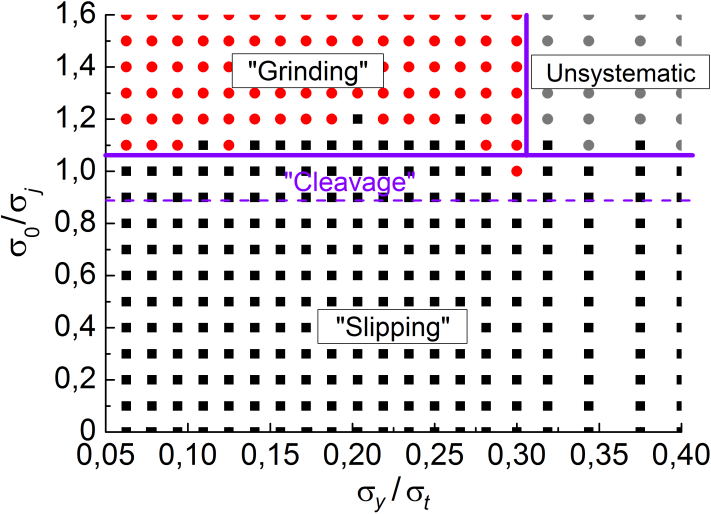


Figure S10. The case study map of asperity interaction modes in terms of two dimensionless material parameters: the ratio of specific attractive force $\sigma_{0}^{*}$ to material’s pure shear strength σ*_j_* (the ordinate axis) and yield stress σ*_y_* normalized to the tensile strength σ*_t_* (the abscissa axis). The map corresponds to materials with von Mises strength (*a*=1) and “reference” values of Young’s and strain hardening moduli.

1. **Study of the mesh size effect**

We have carried out a numerical study to analyze the influence of the mesh size (the size of discrete elements) on the regimes of wear of interacting asperities and the position of the boundary between “high adhesion” regime (shear band driven wear) and “low adhesion regime” (slipping or cleavage). In the framework of this study, we simulated the interaction of asperities, which consist of elements of size *d*=12.5 μm and *d*=6.25 μm. These chosen values of element size are two and four times smaller than original element size *d*=25 μm considered in the paper. In both cases (at *d*=12.5 μm and *d*=6.25 μm) the geometric characteristics of the samples were the same and were as described in the main body of the paper.

The only geometrical differences were the amplitude and period of regular roughness of the contact surfaces of asperities because amplitude and period are proportional to element size (Figure 1 in the main body of the paper). Note that the initial overlap of asperities was also different as it is equal to the distance between adjacent rows of close-packed elements $d/\sqrt{3}$.

We used the same values of density, Young’s modulus, Poisson’s ratio, yield stress and constant strain hardening coefficient of the material as given in Section 2 of the main body of the paper. As in the original study, various values of shear strength σ*_j_* of the material were considered. We considered a particular case *a*=σ*_c_*/σ*_t_*=1, which means that mean stress has no influence on the shear strength of the material (highly ductile materials). For each shear strength value, we varied adhesion stress σ_0_ from zero up to the value of tensile strength of the material σ*_t_*. The results of this study for element size *d*=25 μm are provided in the main body of the paper (Figure 7).

The results of the study showed that in all three cases considered (*d*=25 μm, *d*=12.5 μm and *d*=6.25 μm), the same trend is observed. At low values of adhesion stress σ_0_, asperities slip without breaking (“slipping” mode). When a certain threshold value $\sigma_{0}=\sigma_{0}^{*}$ is reached (it is comparable with the shear strength σ*_j_* of the material), the mode of interaction changes to plastic smoothing (“grinding”). A comparison of the threshold values $\sigma_{0}^{*}$ for the three cases under consideration (three different mesh sizes) shows their close values for all σ*_j_*. The relative difference in the values of $\sigma_{0}^{*}$ is less than 10%, and moreover, it is not systematic. Therefore, in all three cases, the dependences $\sigma_{0}^{*}\left(\sigma_{j}\right)$ are approximated with good accuracy by a single straight line shown in Figure 7 in the main body of the paper (see Figure S11).


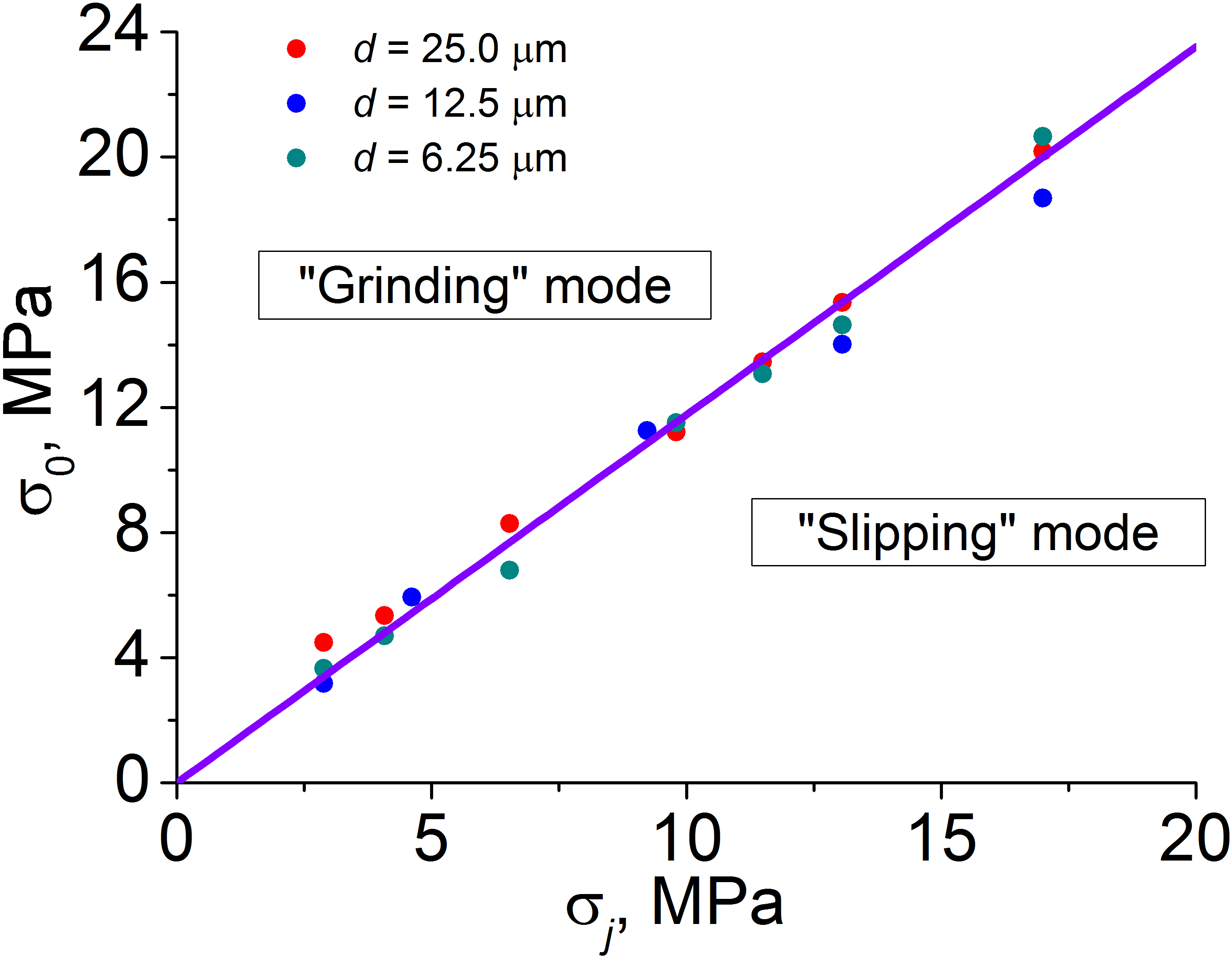


Figure S11. The position of the boundary, which limits the region of “grinding” wear mode from below (slipping or cleavage regime) in the coordinate plane (σ*_j_*, σ_0_), where σ*_j_* is the shear strength of the material, σ_0_ is the specific value of attractive force between spatially separated surfaces. Points show threshold adhesion stresses $\sigma_{0}^{*}$ at corresponding values of material shear strength σ*_j_*. Different colors of points correspond to different values of element size *d*. Violet solid line is the same as in Figure 7 of the main body of the paper. The plot corresponds to highly ductile materials with von Mises strength criterion (*a*=1) and “reference” values of Young’s modulus and yield stress.

We suppose that insignificant/nonprincipal difference in the values of $\sigma_{0}^{*}$ results from the above-mentioned difference in the values of amplitude and period of regular roughness of asperity surface as well as from the difference in initial overlap of asperities (all mentioned parameters are proportional to the element size).

**References**

1. Dmitriev, A.I. & Österle, W. Modelling the sliding behavior of tribofilms forming during automotive braking: impact of loading parameters and property range of constituents. *Tribol. Lett.* **53**, 337-351 (2014).
2. Dmitriev, A.I., Kuznetsov, V.P., Nikonov, A.Yu. & Smolin, I.Yu. Modeling of nanostructuring burnishing on different scales. *Phys. Mesomech.* **17**, 243-249 (2014).
3. Popov, V.L. & Dimaki, A.V. Friction in an adhesive tangential contact in the Coulomb-Dugdale approximation. *The J. Adhesion* **93**, 1131-1145 (2017).
4. Dimaki, A., Shilko, E., Psakhie, S. & Popov, V. Simulation of fracture using a mesh-dependent fracture criterion in the discrete element method. *Facta Univ. Mech. Engng.* **16**, 41-50 (2018).
5. Liu, T., Jagota, A. & Hui C.-Y. Adhesive contact of a rigid circular cylinder to a soft elastic substrate – the role of surface tension. *Soft Matter* **11**, 3844 (2015).
6. von Lautz, J., Pastewka, L., Gumbsch, P. & Moseler, M. Molecular dynamic simulation of collision-induced third-body formation in hydrogen-free diamond-like carbon asperities. *Tribol. Lett.* **63**, 26 (2016).
7. Brink, T. & Molinari, J.-F. Adhesive wear mechanisms in the presence of weak interfaces: Insights from an amorphous model system. *Phys. Rev. Mat.* **3**, 053604 (2019).
8. Rabinowicz, E. The effect of size on the looseness of wear fragments. *Wear* **2**, 4-8 (1958).
9. Aghababaei, R., Warner, D.H. & Molinari, J.-F. Critical length scale controls adhesive wear mechanisms. *Nat. Commun.* **7**, 11816 (2016).
10. Molinari, J.-F., Aghababaei, R., Brink, T., Frérot, L. & Milanese, E. Adhesive wear mechanisms uncovered by atomistic simulations. *Friction* **6**, 245-259 (2018).
